# Keypoint-MoSeq: parsing behavior by linking point tracking to pose dynamics

**DOI:** 10.1038/s41592-024-02318-2

**Published:** 2024-07-12

**Authors:** Caleb Weinreb, Jonah E. Pearl, Sherry Lin, Mohammed Abdal Monium Osman, Libby Zhang, Sidharth Annapragada, Eli Conlin, Red Hoffmann, Sofia Makowska, Winthrop F. Gillis, Maya Jay, Shaokai Ye, Alexander Mathis, Mackenzie W. Mathis, Talmo Pereira, Scott W. Linderman, Sandeep Robert Datta

**Affiliations:** 1 Department of Neurobiology, Harvard Medical School, Boston, MA USA; 2 Department of Electrical Engineering, Stanford University, Stanford, CA USA; 3 Wu Tsai Neurosciences Institute, Stanford University, Stanford, CA USA; 4 Brain Mind and Neuro-X Institute, School of Life Sciences, Ecole Polytechnique Fédérale de Lausanne (EPFL), Lausanne, Switzerland; 5 Salk Institute for Biological Studies, La Jolla, CA USA; 6 Department of Statistics, Stanford University, Stanford, CA USA

**Keywords:** Neuroscience, Computational biology and bioinformatics, Drosophila, Mouse

## Abstract

Keypoint tracking algorithms can flexibly quantify animal movement from videos obtained in a wide variety of settings. However, it remains unclear how to parse continuous keypoint data into discrete actions. This challenge is particularly acute because keypoint data are susceptible to high-frequency jitter that clustering algorithms can mistake for transitions between actions. Here we present keypoint-MoSeq, a machine learning-based platform for identifying behavioral modules (‘syllables’) from keypoint data without human supervision. Keypoint-MoSeq uses a generative model to distinguish keypoint noise from behavior, enabling it to identify syllables whose boundaries correspond to natural sub-second discontinuities in pose dynamics. Keypoint-MoSeq outperforms commonly used alternative clustering methods at identifying these transitions, at capturing correlations between neural activity and behavior and at classifying either solitary or social behaviors in accordance with human annotations. Keypoint-MoSeq also works in multiple species and generalizes beyond the syllable timescale, identifying fast sniff-aligned movements in mice and a spectrum of oscillatory behaviors in fruit flies. Keypoint-MoSeq, therefore, renders accessible the modular structure of behavior through standard video recordings.

## Abstract (web summary)

Keypoint-MoSeq is an unsupervised behavior segmentation algorithm that extracts behavioral modules from keypoint tracking data acquired with diverse algorithms, as demonstrated in mice, rats and fruit flies. The extracted modules faithfully reflect human-annotated behaviors even though they are obtained in an unsupervised fashion.

## Main

Work from ethology demonstrates that behavior—a chain of actions traced by the body’s movement over time—is both continuous and discrete^1–3^. The rapid advance of keypoint tracking methods (including SLEAP^4^, DeepLabCut^5^ and others^6,7^) has given researchers broad access to the continuous dynamics that underlie animal behavior^8^. But parsing these dynamics into chains of discrete actions remains an open problem^9–11^. While several action segmentation approaches exist^12–17^, their underlying logic and assumptions differ, with different methods often giving distinct descriptions of the same behavior^13,15^. An important gap, therefore, exists between our access to movement kinematics and our ability to understand their underlying structure.

One method for parsing behavior in mice is Motion Sequencing (MoSeq)^16,18–21^. MoSeq uses unsupervised machine learning to transform its inputs—which are not keypoints, but three-dimensional (3D) depth videos—into a set of behavioral motifs (like rears, turns and pauses) called syllables. To identify syllables, MoSeq searches for discontinuities in behavioral data at a timescale that is set by the user; this timescale is specified through a ‘stickiness’ hyperparameter that influences the frequency with which syllables can transition. In the mouse, where MoSeq has been extensively applied, pervasive discontinuities at the sub-second-to-second timescale mark boundaries between syllables, and the stickiness hyperparameter is explicitly set to capture this timescale^16^.

Previous studies have applied MoSeq to characterize the effects of genetic mutations, drugs, neural manipulations and changes in the sensory or physical environment^16,22–24^. MoSeq syllables are encoded in the dorsolateral striatum (DLS)—an area important for action selection—and can be individually reinforced through closed-loop dopamine stimulation^22,23^, arguing that MoSeq-identified syllables are meaningful units of behavior used by the brain to organize action sequences. But MoSeq’s reliance on depth cameras is a substantial constraint; depth cameras are difficult to deploy, suffer from high sensitivity to reflections and have limited temporal resolution^25^. In principle, these limits could be overcome by applying MoSeq to keypoint data. But attempts to do so have thus far failed: researchers applying MoSeq-like models to keypoint data have reported flickering state sequences that switch much faster than the animal’s actual behavior^13^.

Here we confirm this finding and trace its cause to jitter in the keypoint estimates, which is mistaken by MoSeq for behavioral transitions. To address this challenge, we reformulated the model underlying MoSeq to simultaneously infer correct pose dynamics (from noisy or even missing data) and the behavioral syllables they represent. We validate this model, called keypoint-MoSeq, using accelerometry measurements, neural activity recordings and supervised behavior labels from expert observers, and show that it generalizes beyond mouse syllables to capture behaviors at multiple timescales and in several species. Because keypoint tracking can be applied in diverse settings (including natural environments), requires no specialized hardware and affords direct control over which body parts to track and at what resolution, we anticipate that keypoint-MoSeq will serve as a general tool for parsing the structure of behavior. To facilitate broad adoption, we have directly integrated keypoint-MoSeq with widely used tracking methods (including SLEAP and DeepLabCut) and made the code freely accessible for academic users at http://www.moseq4all.org/.

## Results

**Fig. 1:**
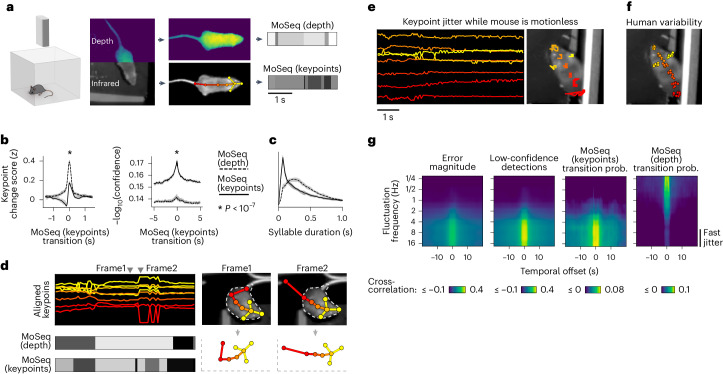
Keypoint trajectories exhibit sub-second structure. **a**, Left: simultaneous depth and 2D infrared (IR) recording setup. Middle: pose representations using the depth data (top) or IR (bottom, tracked keypoints indicated). Right: Example syllable sequences from MoSeq applied to depth data (referred to as ‘MoSeq (depth)’) or to keypoint data (referred to as ‘MoSeq (keypoints)’). Figure created with SciDraw under a CC BY 4.0 license. **b**, Keypoint change scores or low-confidence detection scores, relative to the onset of MoSeq transitions (*x* axis) derived from either depth (gray) or keypoint (black) data. Differences in each case were significant (*P* = 2 × 10^−7^ over *N* = 20 model fits, Mann–Whitney *U* test; plots show mean and range across model fits). **c**, Comparison of syllable durations for MoSeq (keypoints) and MoSeq (depth), showing mean and inter-95% confidence interval range across *N* = 20 model fits. **d**, Left: keypoint detection errors, including high-frequency fluctuations in keypoint coordinates (top row) and error-induced syllable switches (bottom row). Right: keypoint coordinates before (frame1) and during (frame2) an example keypoint detection error. This error (occurring in the tail keypoint) causes a shift in egocentric alignment, hence changes across the other tracked keypoints. **e**, 5-s interval during which the mouse is immobile yet the keypoint coordinates fluctuate. Left: egocentrically aligned keypoint trajectories. Right: path traced by each keypoint during the 5-s interval. **f**, Variability in keypoint positions assigned by eight human labelers. **g**, Cross-correlation between various features and keypoint fluctuations at a range of frequencies. Each heat map represents a different scalar time series (such as ‘transition probability’—the likelihood of a syllable transition on each frame). Each row shows the cross-correlation between that time series and the time-varying power of keypoint fluctuations at a given frequency.

Mouse syllables are evident in depth-based video recordings as discontinuities of movement that reoccur with sub-second cadence^16^. To test if the same sub-second structure is present in keypoint data, we recorded conventional videos of mice exploring an open field arena and used a neural network to track eight keypoints (two ears and six points along the dorsal midline). We also captured simultaneous depth videos for comparison to depth-based MoSeq (Fig. 1a).

Similar sub-second discontinuities appeared in both the depth and keypoint data, with a keypoint-based change score (total velocity of keypoints after egocentric alignment) spiking at the transitions between depth-based MoSeq syllables (Fig. 1b). Yet when we applied MoSeq directly to the keypoint data, it failed to recognize these discontinuities as syllable transitions, instead generating implausibly brief syllables that aligned poorly with the keypoint change score (Fig. 1b,c). These observations are consistent with prior work showing that MoSeq underperforms alternative clustering methods when applied to keypoints^13,26^.

**Extended Data Fig. 1:**
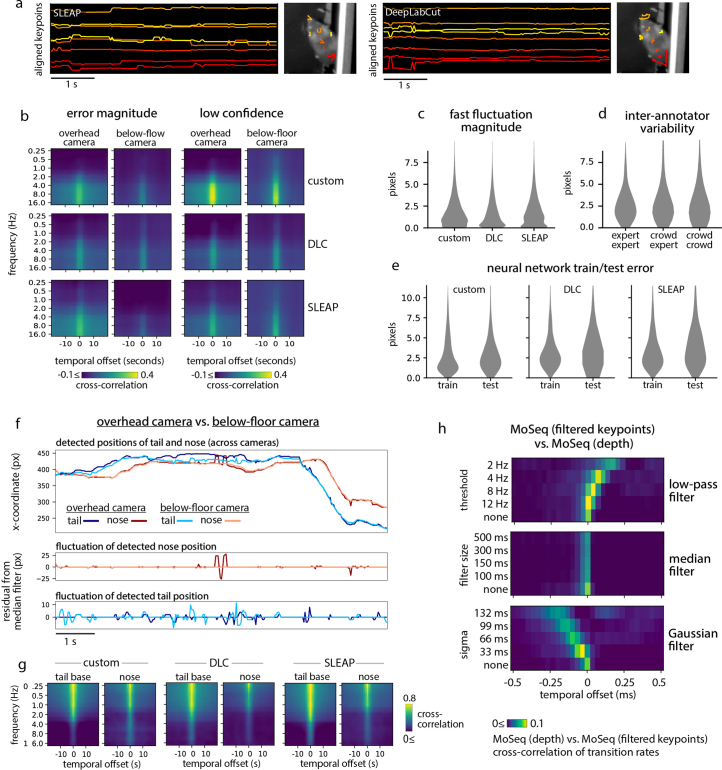
Markerless pose tracking exhibits fast fluctuations that are independent of behavior yet affect MoSeq output. **a)** Example of a 5-second interval during which the mouse is still yet the keypoint coordinates fluctuate, as shown in Fig. 1e, but here for SLEAP and DeepLabCut respectively. **Left:** egocentrically aligned keypoint trajectories. **Right:** path traced by each keypoint during the 5-second interval. **b)** Cross-correlation between the spectral content of keypoint fluctuations and either error magnitude (left) or a measure of low-confidence keypoint detections (right). **c)** Magnitude of fast fluctuations in keypoint position for three different tracking methods, calculated as the per-frame distance from the detected trajectory of a keypoint to a smoothened version of the same trajectory, where smoothing was performed using a gaussian kernel with width 100ms (N=4 million keypoint detections). **d)** Inter-annotator variability, shown as the distribution of distances between multiple annotations of the same keypoint. Annotations were either crowd-sourced or obtained from experts (N=200 frames and N=4 labelers). **e)** Train- and test- error distributions for each keypoint tracking method (N=800 held out keypoint annotations). **f) Top:** position of the nose and tail-base over a 10-second interval, shown for both the overhead and below-floor cameras. **Bottom:** fast fluctuations in each coordinate, obtained as residuals after median filtering. **g)** Cross-correlation between spectrograms obtained from two different camera angles for either the tail base or the nose, shown for each tracking method. **h)** Cross-correlation of transitions rates, comparing MoSeq applied to depth and MoSeq applied to keypoints with various levels of smoothing using a low-pass, Gaussian, or median filter (N=1 model fit per filtering parameter).

We wondered whether this poor performance could be explained by noise in the keypoint data, which might introduce subtle discontinuities that are falsely recognized by MoSeq as behavioral transitions. In our data, this noise took the form of high-frequency jitter that reflected errors in body part detection or rapid jumps in the inferred location of a stationary body part (Fig. 1d,e, Extended Data Fig. 1a,b and Supplementary Video 1). Much of the jitter—which was pervasive across camera angles and tracking methods—seemed to reflect inherent ambiguity in the true location of a keypoint, as frame-to-frame fluctuations in detected keypoint position had a similar scale as the variability in human labeling (Fig. 1f and Extended Data Fig. 1b–e). We confirmed that the jitter was unrelated to true movement by tracking the same body part using multiple cameras; although overall movement trajectories were almost identical across cameras, high-frequency fluctuations around those trajectories were uncorrelated, suggesting that the fluctuations are a tracking artifact (Extended Data Fig. 1f,g).

Consistent with the possibility that keypoint noise dominates MoSeq’s view of behavior, syllable transitions derived from keypoints—but not depth—frequently overlapped with jitter and low-confidence estimates of keypoint position (Fig. 1b,g). We were unable to correct this defect through simple smoothing: application of a low-pass filter—while removing jitter—also blurred true transitions, preventing MoSeq from identifying syllable boundaries (Extended Data Fig. 1h). Median filtering and Gaussian smoothing also yielded no improvement. These data reveal that high-frequency tracking noise prevents MoSeq from accurately segmenting behavior.

### Hierarchical modeling decouples noise from behavior

**Fig. 2:**
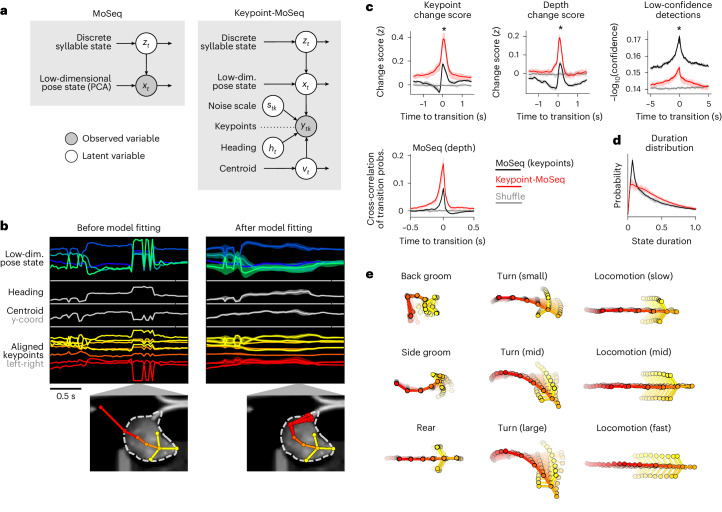
Hierarchical modeling of keypoint trajectories decouples noise from pose dynamics. **a**, Graphical models illustrating traditional MoSeq and keypoint-MoSeq. In both models, a discrete syllable sequence governs pose dynamics in a low-dimensional pose state; these pose dynamics are either described using principal component analysis (PCA; as in ‘MoSeq’; left) or inferred from keypoint observations in conjunction with the animal’s centroid and heading, as well as a noise scale that discounts keypoint detection errors (as in ‘keypoint-MoSeq’; right). **b**, Example of error correction by keypoint-MoSeq. Left: before fitting, all variables (*y* axis) are perturbed by incorrect positional assignment of the tail-base keypoint (whose erroneous location is shown in the bottom inset). Right: Keypoint-MoSeq infers plausible trajectories for each variable (shading represents the 95% confidence interval). The inset shows several likely keypoint coordinates for the tail-base inferred by the model. **c**, Top: various features averaged around syllable transitions from keypoint-MoSeq (red) versus traditional MoSeq applied to keypoint data (black), showing mean and inter-95% confidence interval range across *N* = 20 model fits. Bottom: cross-correlation of syllable transition probabilities between each model and depth MoSeq. Shaded regions indicate bootstrap 95% confidence intervals. Peak height represents the relative frequency of overlap in syllable transitions. Differences in each case were significant (*P = 2 × 10^−7^ over *N* = 20 model fits, Mann–Whitney *U* test). **d**, Duration distribution of the syllables from each of the indicated models. Shading as in **c**. **e**, Average pose trajectories for example keypoint-MoSeq syllables. Each trajectory includes ten poses, starting 165 ms before and ending 500 ms after syllable onset.

Keypoint jitter contaminates MoSeq syllables because MoSeq assumes that each keypoint is a faithful representation of a point on the animal, and thus cannot distinguish noise from real behavior. To address this issue, we rebuilt MoSeq as a switching linear dynamical system (SLDS)—a class of model that explicitly disentangles signal from noise in time-series data^27,28^. This model—called ‘keypoint-MoSeq’—has three hierarchical levels (Fig. 2a): a discrete state sequence that governs trajectories in a low-dimensional pose space, which then combines with location and heading information to yield actual keypoint coordinates. When fit to data, keypoint-MoSeq estimates for each frame the animal’s location and pose, the noise in each keypoint^29^ and the identity of the current behavioral syllable (Fig. 2a). Because of its structure, when a single keypoint implausibly jumps from one location to another, the model can attribute this sudden displacement to noise and preserve a smooth pose trajectory; if all the keypoints suddenly rotate within the egocentric reference frame, the model can adjust the inferred heading for that frame and restore a plausible sequence of coordinates (Fig. 2b).

**Extended Data Fig. 2:**
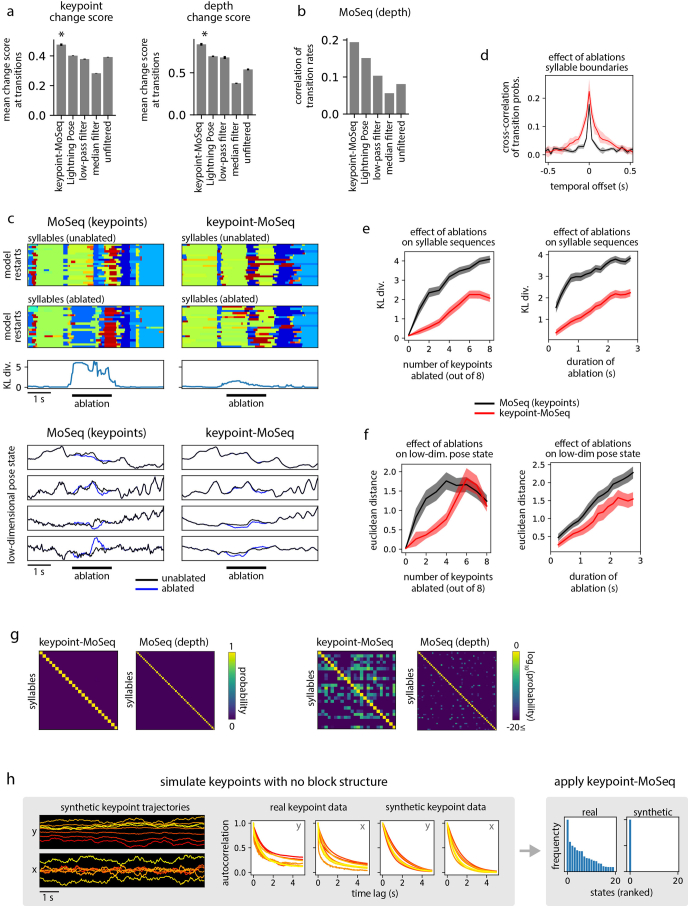
Keypoint-MoSeq is robust to noise and missing data. **a)** Mean change score values at syllable transitions. Syllables were either derived from keypoint-MoSeq applied to (unfiltered) keypoints from our custom neural network, or from traditional MoSeq applied to several versions of the keypoint data, including keypoints inferred from Lightning Pose, or keypoints from our custom neural network followed by low-pass filtering, median filtering, or no filtering. Error bars show standard deviation across N=20 model fits. The change scores are highest for keypoint-MoSeq (P < 10^−4^ over N=20 model fits, Mann-Whitney U test). **b)** Correlations of transition probabilities (that is, the probability of a new syllable starting at each frame), comparing depth MoSeq with each of the keypoint models shown in (a). **c)** Example of model responses to a one-second-long ablation of keypoint observations, shown for keypoint-MoSeq (right) and traditional AR-HMM-based MoSeq (left). **Top: Change in syllable sequences**. Each heatmap row represents an independent modeling run and each column represents a frame. The set of labels on each frame define a distribution, and the Kullback-Leibler divergence (KL div.) between the ablated and unablated distributions is plotted below. **Bottom: Change in low-dimensional pose state**. Estimated pose trajectories derived from unablated (black) or ablated (blue) data. Each dimension of the latent pose space is plotted separately. Lines reflect the mean across modeling runs. **d)** Cross-correlation of transition probabilities for ablated vs. unablated data (computed over frames that were included in an ablation), shown for keypoint-MoSeq (red) and traditional AR-HMM-based MoSeq (red), Shading shows bootstrap 95% confidence intervals for N=20 model fits. Solid line shows cross-correlation using all N=20 models (without bootstrapping). **e)** Mean Kullback-Leibler divergence [as described in (c)] across all ablation intervals, stratified by number of ablated keypoints (left) or duration of the ablation (right). Shading represents the 99% confidence interval of the mean. **f)** Mean distance between pose states estimated from ablated vs. unablated data, with colors and shading as in (e). **g)** Syllable cross-likelihoods, defined as the probability, on average, that time-intervals assigned to one syllable (column) could have arisen from another syllable (row). Cross-likelihoods were calculated for keypoint-MoSeq and for depth MoSeq. The results for both methods are plotted twice, using either an absolute scale (left) or a log scale (right). **h)** Modeling results for synthetic keypoint data with a similar statistical structure as the real data but lacking in changepoints. **Left:** example of synthetic keypoint trajectories. **Middle:** autocorrelation of keypoint coordinates for real vs. synthetic data, showing similar dynamics at short timescales. **Right:** distribution of syllable frequencies for keypoint-MoSeq models trained on real vs. synthetic data.

Unlike traditional MoSeq, keypoint-MoSeq homed in on behavioral syllables rather than noise in the keypoint data, yielding syllable transitions that overlapped more strongly with changepoints in pose, correlated better with syllable transitions from depth MoSeq and clustered less around low-confidence neural network detections (Fig. 2c). Keypoint-MoSeq also outperformed traditional MoSeq when the latter was applied to filtered keypoint data, or to keypoints inferred with a pose estimation method (Lightning Pose) that includes a jitter penalty in its training objective (Extended Data Fig. 2a,b). Furthermore, when we simulated missing data by ablating subsets of keypoints within random (0–3 s) intervals, keypoint-MoSeq was better able to preserve syllable labels and boundaries than traditional MoSeq (Extended Data Fig. 2c–f). From a modeling perspective, the output of MoSeq was sensible: cross-likelihood analysis revealed that keypoint-based syllables were mathematically distinct trajectories in pose space, and submitting synthetic keypoint data that lacked any underlying block structure to keypoint-MoSeq resulted in models that failed to identify distinct syllables (Extended Data Fig. 2g,h).

**Extended Data Fig. 3:**
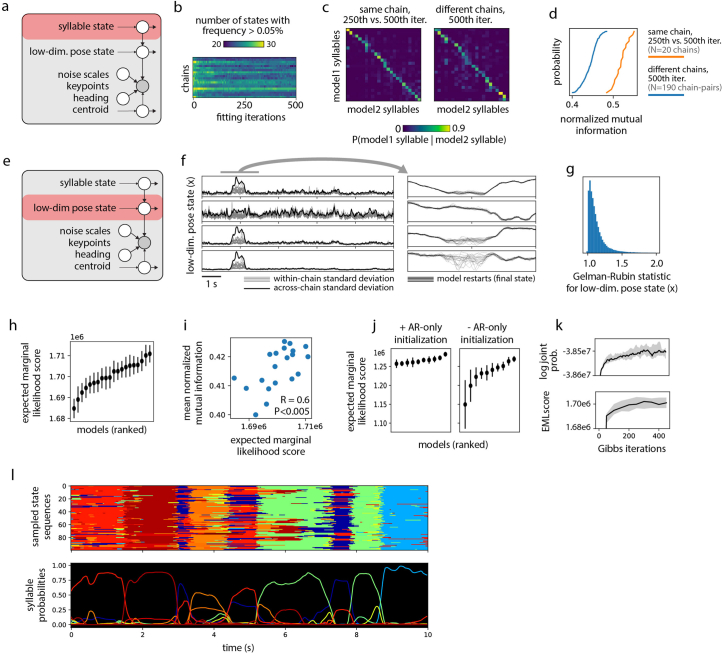
Convergence and model selection. **a)** Probabilistic graphical model (PGM) for keypoint-MoSeq highlighting the discrete syllable state. **b)** Number of syllables identified by keypoint-MoSeq as a function of fitting iteration, shown for multiple independent runs of fitting (referred to as ‘chains’). **c)** Confusion matrices depicting closer agreement between syllables from the same chain at different stages of fitting (left) compared to syllables from different chains at the final stage of fitting (right). **d)** Distributions of syllable sequence similarity [quantified by normalized mutual information (NMI)], either within chains at different iterations (N=20) or across chains (N=190). **e)** PGM highlighting pose state. **f) Left:** within- and between- chain variation in pose state, shown for each dimension of pose (rows) across an example 10-second interval. Gray lines represent the variation across fitting iterations within each chain, and black lines represent the total variation across chains and fitting iterations. **Right:** zoom-in on a 2-second interval showing close agreement in the final pose trajectory learned by each chain. **g)** Distribution of the Gelman-Rubin statistic (ratio of within-chain variance to total variance) across timepoints and dimensions of the pose state. **h)** Expected marginal likelihood (EML) scores (defined as a mean over marginal likelihoods) for the final model parameters learned by each chain. Vertical bars represent standard error based on N=20 chains. **i)** The scores shown in (h) correlate with mean NMI for each model, which is low when a model’s syllable sequences are dissimilar from those of other models (P=0.005, Pearson test). **j)** EML scores are higher for models fit with an autoregressive-only (AR-only) initialization stage (left) compared to those without (right; P = 0.004, N=20 fits for each method, Mann-Whitney U test). Plotted as in (h). **k)** EML scores (bottom) plateau within 500 iterations of Gibbs sampling and have a similar trajectory as the model log joint probability (top). Black lines represent the median across N=20 chains and shaded regions represent inter-quartile interval. **l)** Illustration of uncertainty in syllable sequence given a fixed set of syllable definitions. **Top:** syllable sequences derived from Gibbs sampling (conditioning on fixed autoregressive parameters and transition probabilities), shown for an example 10-second window. **Bottom:** per-frame marginal probability estimates for each syllable. Each line is one syllable, with colors as in the heatmap above.

Because keypoint-MoSeq produces slightly different syllable segmentations when run multiple times with different random seeds, we developed a likelihood-based metric that allows post hoc ranking of model runs (Extended Data Fig. 3a–g); the metric tends to be lowest for outlier models and highest for those that are consensus-like, providing a rational basis for model selection (Extended Data Fig. 3h–k). The metric revealed that 500 fitting iterations (~30 min of compute time on a GPU for ~5 h of data) are sufficient to achieve a good model fit with our open field dataset. Rather than choosing a single best model, users can also estimate an approximate probability distribution over syllable labels, although full Bayesian convergence remains impractical (Extended Data Fig. 3l).

**Extended Data Fig. 4:**
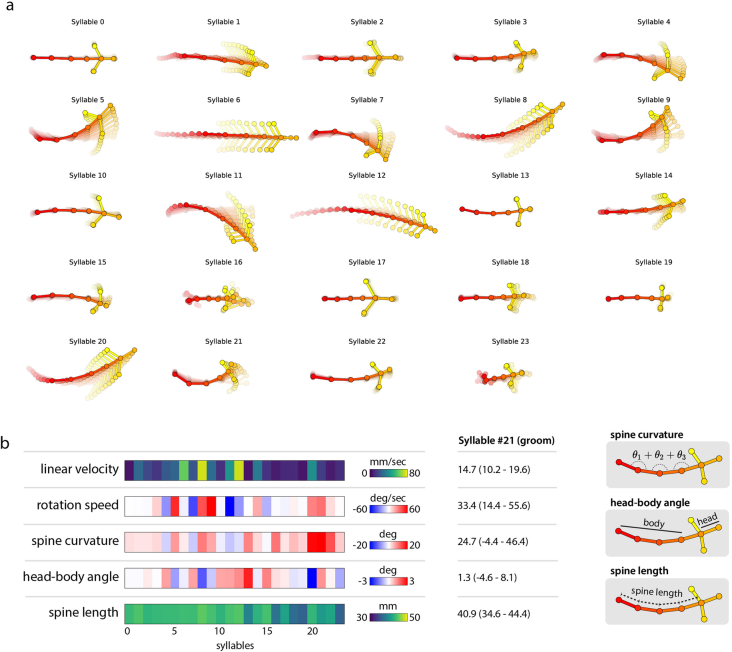
Behaviors captured by keypoint-MoSeq syllables. **a)** Average pose trajectories for syllables identified by keypoint-MoSeq. Each trajectory includes ten evenly timed poses from 165ms before to 500ms after syllable onset. **b) Kinematic and morphological parameters for each syllable. Left:** Average values of five parameters (rows) for each syllable (column). **Middle:** Mean and interquartile range of each parameter for one example syllable. **Right:** cartoons illustrating the computation of the three morphological parameters.

In our open field data, keypoint-MoSeq identified 25 syllables that were easily distinguishable to human observers (Extended Data Fig. 4a and Supplementary Videos 2 and 3). These included categories of behavior (for example, rearing, grooming and walking), and variations within categories (for example, turn angle, speed; Fig. 2e). Importantly, keypoint-MoSeq preserved access to the kinematic and morphological parameters that underlie each behavioral syllable (Extended Data Fig. 4b). Thus, keypoint-MoSeq can provide an interpretable segmentation of behavior from standard two-dimensional (2D) keypoint tracking data.

### Keypoint-MoSeq is sensitive to behavioral transitions

To characterize keypoint-MoSeq, we related the discovered syllables to orthogonal measures of behavior and neural activity and compared them to the behavioral states identified by alternative behavior analysis methods. These alternatives, which include VAME, MotionMapper and B-SOiD, all work by transforming keypoint data into a feature space that reflects the local dynamics around each frame, and then clustering frames according to those features^12,13,17,30^.

**Fig. 3:**
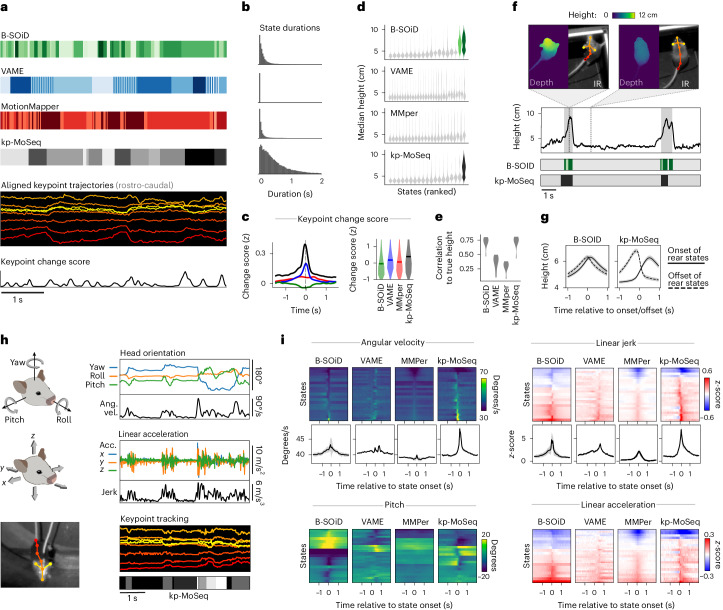
Keypoint-MoSeq captures the temporal structure of behavior. **a**, Output from four methods applied to the same 2D keypoint dataset. **b**, Distribution of state durations for each method in **a**. **c**, Left: average keypoint change scores (*z*-scored) around transitions identified by each method. Right: distribution of change scores at the transition point (‘MMper’ refers to MotionMapper). **d**, Distribution of mouse heights (measured by depth camera) for each unsupervised behavior state. States are classified as rear specific (and given a non-gray color in the plot) if they have median height > 6 cm. **e**, Accuracy of models trained to predict mouse height from behavior labels showing the distribution of accuracies across *N* = 10 recordings. **f**, Bottom: state sequences from keypoint-MoSeq and B-SOiD during a pair of example rears. States are colored as in **d**. Top: mouse height over time with rears shaded gray. Callouts show depth and IR views of the mouse during two example frames. **g**, Mouse height aligned to the onsets (solid lines) or offsets (dashed lines) of rear-specific states defined in **d**, showing mean and 95% confidence of the mean. **h**, Signals captured from a head-mounted IMU, including absolute 3D head orientation (top) and relative linear acceleration (bottom). Each signal and its rate of change, including angular velocity (ang. vel.) and jerk (the derivative of acceleration), are plotted during a 5-s interval. Figure created with SciDraw under a CC BY 4.0 license. **i**, IMU signals aligned to the onsets of each behavioral state. Each heat map row represents a state. Line plots show the median across states for angular velocity and jerk (average and standard across *N* = 10 model fits). Keypoint-MoSeq peaks at a higher value for both signals (*P* < 0.0005, *N* = 10, Mann–Whitney *U* test).

**Extended Data Fig. 5:**
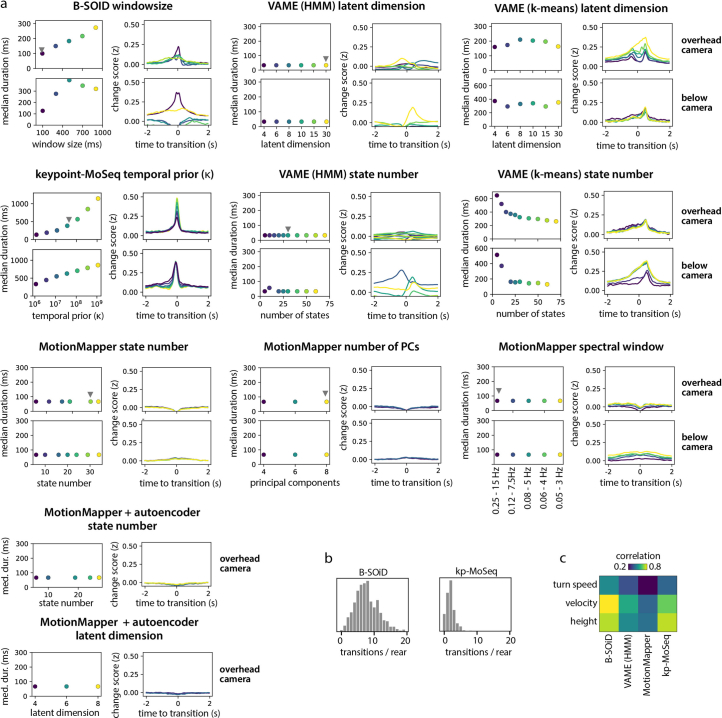
Method-to-method differences in sensitivity to behavioral changepoints are robust to parameter settings. **a)** Output of unsupervised behavior segmentation algorithms across a range of parameter settings, applied to 2D keypoint data from two different camera angles (N=1 model fits per parameter set). The median state duration (left) and the average (z-scored) keypoint change score aligned to state transitions (right) are shown for each method and parameter value. Gray pointers indicate default parameter values used for subsequent analysis (see Supplementary Table 3 for a summary of parameters). **b)** Distributions showing the number of transitions that occur during each rear. **c)** Accuracy of kinematic decoding models that were fit to state sequences from each method.

When applied to our open field data, behavioral states from VAME, B-SOiD and MotionMapper were usually brief (median duration 33–100 ms, compared to ~400 ms for keypoint-MoSeq) and their transitions aligned poorly with changepoints in keypoint data, suggesting diminished sensitivity to the natural breakpoints in mouse behavior (Fig. 3a–c). This observation was not parameter dependent, because it remained true across a broad range of temporal windows (used by B-SOiD and MotionMapper) and after comprehensive scans over latent dimension, state number, clustering mode and preprocessing options (across all methods as applicable; Extended Data Fig. 5a).

Rearing offers a clear example of the differing sensitivity of each method to temporal structure. B-SOiD and keypoint-MoSeq both learned a specific set of rear states, and each encoded the mouse’s height with comparable accuracy (Fig. 3d,e). Yet the rear states had different dynamics. Whereas keypoint-MoSeq typically detected two syllable transitions per rear (one entering the rear and one exiting), B-SOiD detected five to ten different transitions per rear, including switches between distinct rear states as well as flickering between rear and non-rear states (Fig. 3f and Extended Data Fig. 5b). Whereas mouse height increased at transitions into keypoint-MoSeq’s rear state and fell at transitions out of it, height tended to peak symmetrically at transitions into and out of B-SOiD’s rear states (Fig. 3g); this observation suggests that—at least in this example—B-SOiD does not effectively identify the boundaries between syllables, but instead fragments them throughout their execution.

**Extended Data Fig. 6:**
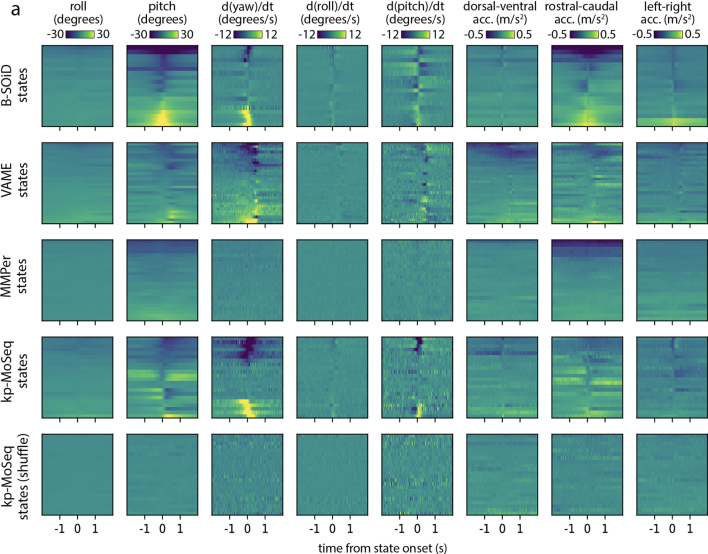
Accelerometry reveals kinematic transitions at the onsets of keypoint-MoSeq states. **a)** IMU signals aligned to state onsets from several behavior segmentation methods. Each row corresponds to a behavior state and shows the average across all onset times for that state. A single model fit is shown for each method.

We also evaluated each method using an orthogonal kinematic measurement: 3D head angle and acceleration from head-mounted inertial measurement units (IMUs; Fig. 3h). Behavioral transitions were identifiable in the IMU data as sudden changes in acceleration (quantified by jerk) and orientation (quantified by angular velocity). These measures tended to overlap with state transitions from keypoint-MoSeq but less so (or not at all) for B-SOiD, MotionMapper and VAME (Fig. 3i). Furthermore, IMU-extracted behavioral features (like head pitch or acceleration) typically rose and fell symmetrically around B-SOiD, MotionMapper and VAME-identified transitions, while keypoint-MoSeq identified asymmetrical changes in these features (Fig. 3i and Extended Data Fig. 6a).

The fact that keypoint-MoSeq more clearly identifies behavioral boundaries does not necessarily mean that it is better at capturing the overall content of behavior. Indeed, coarse kinematic parameters were captured equally well by all four of the tested methods (Extended Data Fig. 5c). However, the fact that movement parameters—as measured by accelerometry—change suddenly at the onset of keypoint-MoSeq syllables, but not at the onset of B-SOiD, VAME or MotionMapper states, provides evidence that these methods afford fundamentally different views of temporal structure in behavior.

### State transitions align with fluctuations in neural data

**Fig. 4:**
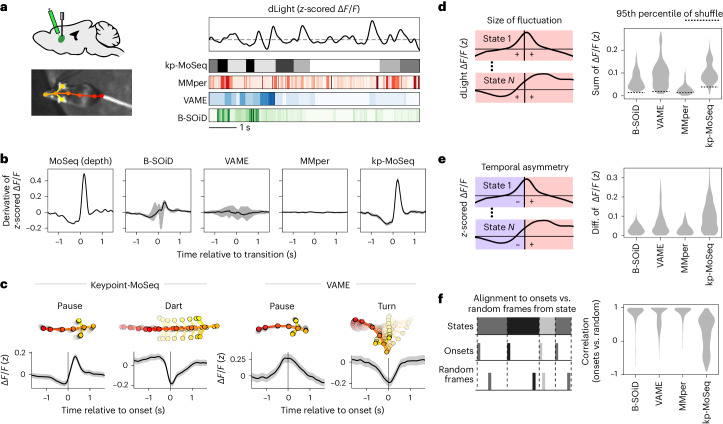
Keypoint-MoSeq syllable transitions align with fluctuations in striatal dopamine. **a**, Illustration depicting simultaneous recordings of dopamine fluctuations in the DLS obtained from fiber photometry (top) and unsupervised behavioral segmentation of 2D keypoint data (bottom). Adapted from ref. ^22^, Springer Nature Limited. **b**, Derivative of the dopamine signal aligned to state transitions from MoSeq (depth) and each keypoint-based method, showing the mean and 95% confidence of the mean. The derivative peaks at a higher value for keypoint-MoSeq compared to the non-MoSeq methods (*P* < 10^−5^, *N* = 20 model fits per method, Mann–Whitney *U* test). **c**, Average dopamine signal (*z*-scored change in fluorescence, Δ*F/F*) aligned to the onset of example states identified by keypoint-MoSeq and VAME. Shading marks the 95% confidence interval around the mean. **d**, Distributions capturing the magnitude of state-associated dopamine fluctuations across states from each method (merging *N* = 20 model fits per method), where magnitude is defined as the mean total absolute value in a 1-s window centered on state onset. Box plots show median and interquartile range (IQR). **e**, Distributions capturing the temporal asymmetry of state-associated dopamine fluctuations, where asymmetry is defined as the difference in mean dopamine signal during 500 ms after versus 500 ms before state onset. Keypoint-MoSeq syllables have a higher asymmetry score on average than those from other methods (*P* < 10^−4^, *N* = 20 model fits per method, Mann–Whitney *U* test). **f**, Temporal randomization affects keypoint-MoSeq-identified neurobehavioral correlations, but not those identified by other methods. Top: schematic of randomization. The dopamine signal was aligned either to the onsets of each state, as in **c**, or to random frames throughout the execution of each state. Bottom: distributions capturing the correlation of state-associated dopamine fluctuations before versus after randomization. Keypoint-MoSeq syllables have a lower correlation on average than those from other methods (*P* < 10^−4^, *N* = 20 model fits per method, Mann–Whitney *U* test).

A core use case for unsupervised behavioral classification is to understand how the brain generates self-motivated behaviors outside a rigid task structure^9^; in this setting, boundaries between behavioral states serve as surrogate timestamps for alignment of neural data. For example, we recently used depth MoSeq to show that dopamine fluctuations in DLS are temporally aligned to syllable transitions during spontaneous behavior^22^. Here we asked whether the same result was apparent in keypoint-based segmentations of behavior (Fig. 4a).

**Extended Data Fig. 7:**
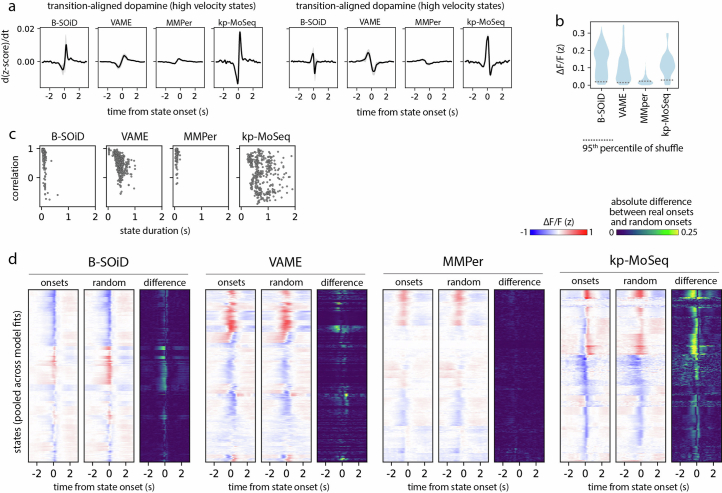
Striatal dopamine fluctuations are enriched at keypoint-MoSeq syllable onsets. **a)** Derivative of the dopamine signal aligned to the onsets of high velocity or low velocity behavior states. States from each method were classified evenly as high or low velocity based on the mean centroid velocity during their respective frames. Plots show mean and inter-95% range across N=20 model fits. **b)** Distributions capturing the average absolute value of the dopamine signal across states from each method. **c)** Relationship between state durations and correlations from Fig. 5f. **d)** Average dopamine fluctuations aligned to state onsets (left) or aligned to random frames throughout the execution of each state (middle), as well as the absolute difference between the two alignment approaches (right), shown for each unsupervised behavior segmentation approach.

Syllable-associated dopamine fluctuations (as captured by dLight photometry) were remarkably similar between depth MoSeq and keypoint-MoSeq, but much lower in amplitude (or nonexistent) when assessed using B-SOiD, VAME and MotionMapper (Fig. 4b and Extended Data Fig. 7a). We wondered if this apparent discrepancy in syllable-associated dopamine could be explained by differences in how each method represents the temporal structure of behavior. If, as we have shown, B-SOiD, VAME and MotionMapper can capture the content of behavior but not the timing of transitions, then average dopamine levels should vary consistently across their behavior states but lack clear dynamics (increases or decreases) at state onsets. Indeed, for all four methods, almost every state was associated with a consistent above-average or below-average dopamine level (Fig. 4c,d and Extended Data Fig. 7b), and yet dopamine dynamics varied widely. Whereas dopamine usually increased at the initiation of keypoint-MoSeq syllables, it was usually flat (having just reached a peak or nadir) at state onsets identified by alternative methods (Fig. 4c–e). Furthermore, aligning the dopamine signal to randomly sampled times throughout the execution of each behavioral state—rather than its onset—altered state-associated dopamine dynamics for keypoint-MoSeq, but made little difference for alternative methods (Fig. 4f and Extended Data Fig. 7c,d). These results suggest that keypoint-MoSeq syllable onsets are meaningful landmarks for neural data analysis, while state onsets identified by alternative methods are often functionally indistinguishable from random timepoints during a behavior.

### Keypoint-MoSeq generalizes across experimental setups and behaviors

**Extended Data Fig. 8:**
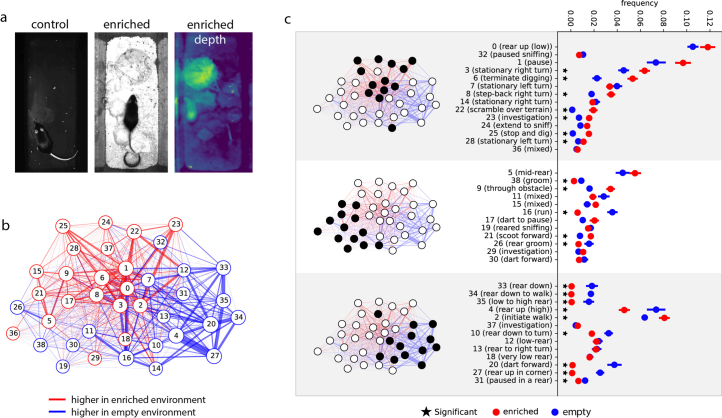
Changes in behavior caused by environmental enrichment. **a)** Example frames from conventional 2D videos of the empty bin (left), and enriched environment (middle), as well as depth video of the enriched environment (right). **b)** Graph showing changes in syllable-to-syllable transition statistics across environments. Edge color and width indicate the sign and magnitude of change in the frequency of each syllable pair. **c) Right:** changes in syllable frequency across environments, with stars indicating significant differences (P < 0.05, N=16, Mann-Whitney U test). Error bars show standard error of the mean. **Left:** Syllable groupings defined by clustering of the transition graph shown in (b).

Keypoint tracking is a powerful means of pose estimation because it generalizes widely across experimental setups. To test whether keypoint-MoSeq inherits this flexibility, we asked if it could quantify changes in behavior induced by environmental enrichment. Mice were recorded in either an empty arena or one that contained bedding, chew toys and a transparent shelter (Extended Data Fig. 8a). The enriched environment was too complex for traditional depth MoSeq but yielded easily to keypoint-based pose estimation. Based on these poses, keypoint-MoSeq identified 39 syllables, of which 21 varied between environments: syllables upregulated in the enriched environment tended to involve manipulation and orientation toward nearby affordances (for example, ‘investigation’, ‘stationary right turn’ and ‘stop and dig’), whereas those upregulated in the empty box were limited to locomotion and rearing (‘dart forward’ and ‘rear-up in corner’; Extended Data Fig. 8b,c). These results suggest that keypoint-MoSeq may be useful in a broad range of experimental contexts, including those whose cluttered structure precludes the effective use of depth cameras.

**Fig. 5:**
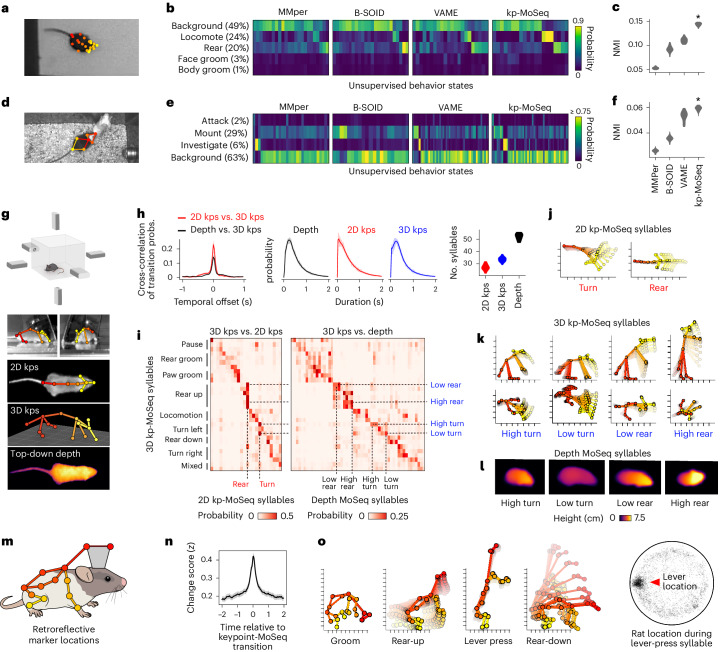
Keypoint-MoSeq generalizes across experimental setups. **a**, Frame from an open field benchmark dataset. **b**, Confusion matrices showing overlap between human-labeled behaviors and unsupervised states. **c**, Normalized mutual information (NMI) between supervised and unsupervised labels, showing the distribution of NMI values across *N* = 20 model fits. Keypoint-MoSeq consistently had higher NMI (*P < 10^−6^, Mann–Whitney *U* test). **d**, Frame from the CalMS21 social behavior benchmark dataset, showing 2D keypoints of the resident mouse. **e**,**f**, Comparison between human labels and unsupervised behavior states of the resident mouse, as in **b** and **c** (*P* < 10^−5^, Mann–Whitney *U* test). **g**, Multi-camera arena for simultaneous recording of 3D keypoints (3D kps), 2D keypoints (2D kps) and depth videos. Figure created with SciDraw under a CC BY 4.0 license. **h**, Comparison of MoSeq outputs from each modality. Left: cross-correlation between 3D transition probabilities and those for 2D keypoints and depth. Shading shows bootstrap 95% confidence intervals; middle: distribution of syllable durations, showing mean and inter-95% confidence interval range across *N* = 20 model fits. Right: number of states with frequency > 0.5%, showing the distribution of state counts across 20 runs of each model. **i**, Overlap of syllables from 2D keypoints (left) or depth (right) with each 3D keypoint-based syllable. **j**–**l**, Average pose trajectories for the syllables marked in **i**. **k**, 3D trajectories are plotted from the side (first row) and top (second row). **l**, Average pose (as depth image) 100 ms after syllable onset. **m**, Location of markers for rat motion capture. Figure created with SciDraw under a CC BY 4.0 license. **n**, Left: average keypoint change score (*z*) aligned to syllable transitions. Shading shows 95% confidence intervals of the mean. Right: durations of keypoint-MoSeq states and inter-changepoint intervals. **o**, Left: pose trajectories of example syllables learned from rat motion capture data. Right: random sample of rat centroid locations during execution of the ‘lever-press’ syllable.

**Extended Data Fig. 9:**
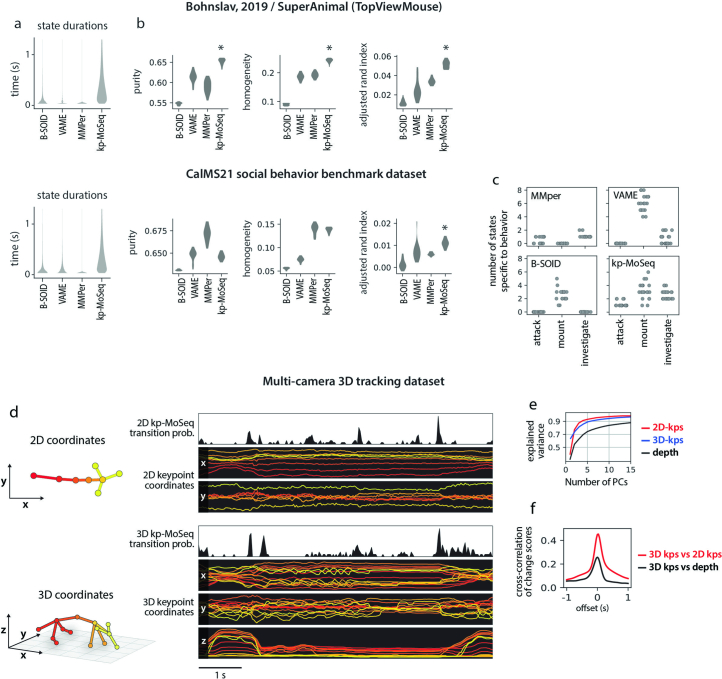
Supervised behavior benchmark. **a)** Distribution of state durations from each behavior segmentation method for the open field benchmark (top) and the CalMS21 social behavior benchmark (bottom). **b)** Three different similarity measures applied to the output of each unsupervised behavior analysis method, showing the median (gray bars) and inter-quartile interval (black lines) across independent model fits (N=20; * P < 10^−5^, for keypoint-MoSeq vs. each other method, Mann-Whitney U test). **c)** Number of unsupervised states specific to each human-annotated behavior in the CalMS21 dataset, shown for 20 independent fits of each unsupervised method. A state was defined as specific if > 50% of frames bore the annotation. **d) Left:** Keypoints tracked in 2D (top) or 3D (bottom) and corresponding egocentric coordinate axes. Right: example keypoint trajectories and transition probabilities from keypoint-MoSeq. Transition probability is defined, for each frame, as the probability of a syllable transition occurring on that frame. **e)** Cumulative fraction of explained variance for increasing number of principal components (PCs). PCs were fit to egocentrically aligned 2D keypoints, egocentrically aligned 3D keypoints, or depth videos respectively. **f)** Cross-correlation between the 3D keypoint change score and change scores derived from 2D keypoints and depth respectively (based on N=20 model fits).

To test if keypoint-MoSeq can also generalize across laboratories—and to better understand the mapping between syllables and human-identified behaviors—we next analyzed a pair of published benchmark datasets^31,32^. The first dataset included human annotations for four mouse behaviors in an open field (locomotion, rearing, face grooming and body grooming) and keypoint detections from the TopViewMouse model in the DLC Model Zoo^33^ (Fig. 5a–c). The second dataset (part of the CalMS21 benchmark^32^) included a set of three manually annotated social behaviors (mounting, investigation and attack) as well as keypoints for a pair of interacting mice (Fig. 5d–f). Keypoint-MoSeq recovered syllables from both datasets whose average duration was ~400 ms, while, as before, the B-SOiD, MotionMapper and VAME identified behavioral states that were much shorter (Extended Data Fig. 9a). Keypoint-MoSeq states also conformed more closely to human-identified behavioral states (Fig. 5c,f and Extended Data Fig. 9b). Although this advantage was modest overall, there were some important differences: in the CalMS21 dataset, for example, MotionMapper, B-SOiD and VAME only identified a single behavior consistently, with B-SOiD and VAME only capturing mounting and MotionMapper only capturing investigation in 100% of model fits; keypoint-MoSeq, in contrast, defined at least one state specific to each of the three behaviors in 100% of model fits (Extended Data Fig. 9c).

The above benchmark datasets differed widely in the number of keypoints tracked (7 for CalMS21 versus 21 for the TopViewMouse model; Fig. 5a,d), raising the question of how the pose representation fed to keypoint-MoSeq influences its outputs. One possibility—suggested by the higher syllable count for depth MoSeq (~50) compared to keypoint-MoSeq fit to 2D keypoints (~25)—is that higher-dimensional input data allows MoSeq to make finer distinctions between behaviors. To test this rigorously, we used multiple cameras to estimate keypoints in 3D (including six keypoints that were not visible in the overhead-camera 2D dataset) and confirmed that the 3D keypoints had higher intrinsic dimensionality than 2D keypoints (Fig. 5g and Extended Data Fig. 9d,e). Despite this difference in dimensionality, similar changepoints were evident in both datasets, and keypoint-MoSeq identified syllables with similarly timed transitions (Fig. 5h and Extended Data Fig. 9f).

There was a bigger change, however, in how behaviors were categorized. Keypoint-MoSeq made finer-grained behavior distinctions based on 3D data as compared to 2D data, especially for behaviors that varied in height (Fig. 5i–l and Supplementary Video 4). Turning, for example, was grouped as a single state based on the 2D keypoint data but partitioned into three states with different head positions based on the 3D keypoint data (nose to the ground versus nose in the air; Fig. 5j–l). Rearing was even more fractionated, with a single 2D syllable splitting six ways based on body angle and trajectory in the 3D keypoint data. Depth-based MoSeq fractionated these behaviors still further. This analysis suggests that higher-dimensional input data permit richer descriptions of behavior, but even relatively low-dimensional 2D keypoint data still capture the timing of behavioral transitions.

### Keypoint-MoSeq parses behavior across species and timescales

To test if keypoint-MoSeq generalizes across rodent species, we analyzed previously published 3D motion capture data derived from rats. In this dataset, rats were adorned with reflective body piercings and recorded in a circular home cage arena with a lever and water spout for operant training (Fig. 5m; Rat7M dataset^34^). As with mice, keypoint-MoSeq syllables aligned with changepoints in the keypoint data (Fig. 5n) and included a diversity of behaviors, including a syllable specific to lever pressing in the arena (Fig. 5o and Supplementary Video 5).

Mice combine postural movements, respiration and whisking to sense their environment. Recent work suggests that rodents coordinate these behaviors in time, generating rhythmic head movements that synchronize with the sniff cycle^35,36^. Using an autoregressive hidden Markov model (AR-HMM), for example, head-movement motifs were discovered that align to respiration and arise during olfactory navigation^21^. Respiration, therefore, defines a fast timescale of mouse behavior that coexists with—but is distinct from—the ~400-ms timescale of behavioral syllables.

**Fig. 6:**
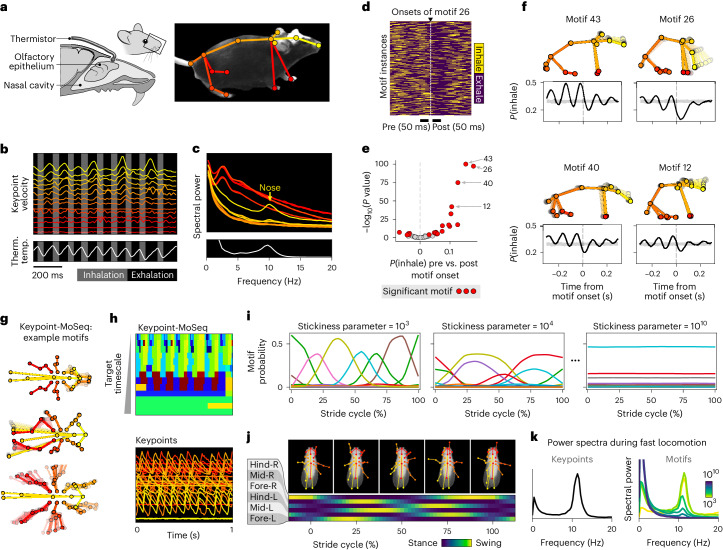
Keypoint-MoSeq segments behavior at multiple timescales. **a**, Setup for recording 3D pose and respiration, including location of thermistor, which monitors temperature fluctuations caused by respiration. Figure created with SciDraw under a CC BY 4.0 license. **b**, 3D keypoint velocities (top) and thermistor signal (bottom) over a 1-s interval. Keypoint traces are colored as in **a** and vertically spaced to ease visualization. **c**, Power spectra of 3D keypoint velocities (top) and thermistor signal (bottom). **d**, Example motif that aligns with inhale-to-exhale transition. The heat map shows respiration states across many instances of the motif. **e**, Volcano plot revealing respiration-aligned motifs. The *x* axis reflects change of inhalation probability during the 50 ms before versus after motif onset. **f**, Keypoint trajectories (top) and motif-aligned inhalation probabilities (bottom) for four motifs highlighted in **e**. Gray shading (bottom) shows the 2.5th-to-97.5th-percentile range of a shuffle distribution. **g**, Average pose trajectories for three fly motifs. **h**, Example of motif sequences during locomotion. Top: Keypoint-MoSeq output for models tuned to a range of timescales. Each row shows the output of a different model. Bottom: Aligned keypoint trajectories (anteroposterior coordinate). **i**, Frequency of motifs across the stride cycle during fast locomotion. Each line corresponds to one motif, and each panel represents a model with a different target timescale. **j**, Top: progression through the stride cycle. Bottom: probability that each leg is in stance or swing phase at each point in the stride; soft boundaries reflect variation in step timing. **k**, Power spectral density of keypoints (left) or motif labels (right) during fast locomotion. Colors in the right-hand plot correspond to models with a range of values for the stickiness hyperparameter, which sets the target timescale.

**Extended Data Fig. 10:**
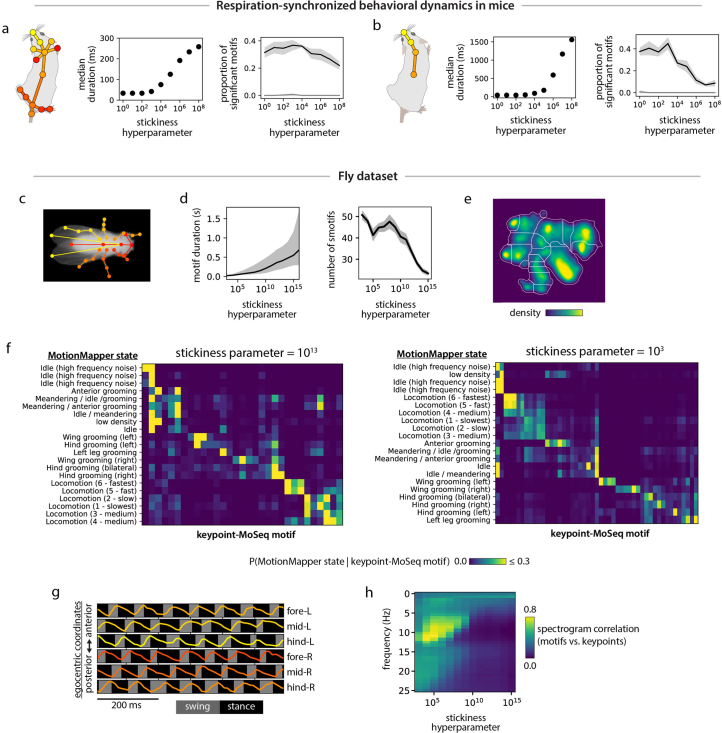
Keypoint-MoSeq identifies behavioral motifs across timescales. **a-b**) Alignment of mouse behavior motifs to respiration. Figure created with SciDraw under a CC BY 4.0 license. **a**) Left: Keypoints used for model fitting. Middle: Median motif durations for models fit with a range of stickiness hyperparameters. Right: Proportion of significantly respiration-aligned motifs, stratified by stickiness hyperparameter, showing mean and standard deviation across N=5 model fits. **b**) As (a), but restricted to upper spine, neck, head, and nose keypoints. **c-h**) Keypoint-MoSeq partitions fly behavior across timescales. **c**) Fly keypoints used for fitting keypoint-MoSeq and MotionMapper. **d**) Motif durations (left) and number of motifs (right) for models trained with a range of target timescales. Ten separate models were fit for each timescale. For motif durations, we pooled the duration distributions from all 20 models and plotted the median duration in black and interquartile range in gray. For motif number, we counted the number of motifs with frequency above 0.5% for each of the 20 models and plotted the mean of this count in black and the standard deviation in gray. **e**) Density of points in 2D ‘behavior space’ generated by MotionMapper. Each white-line delimited region corresponds to a MotionMapper state label. **f**) Confusion matrices showing the frequency of each MotionMapper state during each keypoint-MoSeq motif. **g**) Example of swing and stance annotations over a 600ms window. Lines show the egocentric coordinate of each leg tip (anterior-posterior axis only). Gray shading denotes the swing phase, defined as the interval posterior-to-anterior limb motion. **h**) Cross-correlation between the spectrograms of keypoints and motif labels respectively. Heatmap rows correspond to frequency bands of the spectrograms and columns correspond to models with different target timescales.

To test if keypoint-MoSeq can capture behavioral motifs at this faster timescale, we used 120-Hz cameras to track 3D keypoints of mice and measured respiration with an implanted thermistor^37^ (Fig. 6a). Consistent with prior work, we observed respiration-synchronized fluctuations in nose velocity, although synchrony was weak or absent in other parts of the body (Fig. 6b,c). We then fit keypoint-MoSeq models with a range of target timescales (~35 ms to ~300 ms; Extended Data Fig. 10a). Motifs were defined as ‘respiration coupled’ if they consistently aligned with transitions in respiration state (inhale-to-exhale or exhale-to-inhale; Fig. 6d,e). Although respiration coupling was evident across all models, its prominence peaked at shorter timescales (Extended Data Fig. 10a), especially when fit to a subset of anterior keypoints that emphasized neck and nose movements (Extended Data Fig. 10b). The best-synchronized motifs (from the full-body model) tended to coincide with exhalation and involved isolated movements in which the nose flutters down (Fig. 6e,f). These results suggest that keypoint-MoSeq can characterize fast, sniff-aligned movements in the mouse.

Given that keypoint-MoSeq can parse two different timescales of mouse behavior, we wondered if it could also segment fly behavior, which similarly occurs at multiple well-defined timescales. Flies tend to switch between distinct, oscillatory pose trajectories^17^. These movements can be finely subdivided, as in the coordinated stance and swing phases of locomotion^38^, or more coarsely segmented at the transitions between distinct oscillatory modes (for example, locomotion versus grooming), as they are by MotionMapper^17^. To capture these distinct levels of organization, we fit keypoint-MoSeq to 2D keypoints from flies exploring a flat substrate^17,39^ (Extended Data Fig. 10c). The resulting behavioral motifs varied from tens to hundreds of milliseconds depending on keypoint-MoSeq’s target timescale. At longer timescales, keypoint-MoSeq identified recognizable behaviors such as locomotion, head grooming or left-wing grooming, similarly to the behaviors reported by MotionMapper (Fig. 6g, Supplementary Video 6 and Extended Data Fig 10d–f).

At shorter time scales, keypoint-MoSeq divided these behaviors into their constituent phases. Fast locomotion, for example, was split between six phase-locked motifs that tiled the stride cycle (Fig. 6h). As target timescales grew longer, locomotion merged from six to two phases (corresponding to the alternating swings and stances of a canonical tripod gait) before eventually collapsing to a single motif that encompassed the full stride cycle (Fig. 6h–j and Extended Data Fig. 10g). This shift was evident in the power spectral density of keypoint-MoSeq’s output, which began with a prominent peak at ~12 Hz during fast locomotion (corresponding to the stride cycle) that slowly disappeared as keypoint-MoSeq’s target timescale was increased (Fig. 6k). The same hierarchy of timescales appeared for non-locomotion behaviors as well (Extended Data Fig. 10h). These results demonstrate that keypoint-MoSeq is useful as a tool for fly behavior analysis and suggest a principle for setting its target timescale that depends on whether researchers wish to subdivide the distinct phases of oscillatory behaviors.

## Discussion

Syllables are broadly useful for understanding behavior^16,22–24^, but their scope has been limited by the past requirement for depth data. Here we show that keypoint-MoSeq affords similar insight as depth-based MoSeq while benefiting from the generality of markerless keypoint tracking. Whereas depth MoSeq was limited to a narrow range of spatial scales and frame rates, keypoint-MoSeq can be applied to mammals and insects, parsing behaviors at the second or millisecond timescale. And because keypoint tracking is more robust to occlusion and environmental clutter, it is now possible to parse syllables amid environmental enrichment, in animals behaving alone or socially, with or without headgear and neural implants.

The core innovation enabling keypoint-MoSeq is a probabilistic model that effectively handles occlusions, tracking errors and high-frequency jitter. These noise sources are pervasive in pose tracking^5,26^; because standard methods like SLEAP and DLC process each frame separately, keypoint coordinates tend to jump from frame to frame even when the subject’s pose has not discernably changed. A newer generation of pose tracking methods, such as GIMBAL^29^, Deep Graph Pose^26^ and Lightning Pose^40^, correct for some of these errors; and two-step pipelines that build on these methods may be less prone to keypoint jitter. Here, we describe a different solution: combining noise-correction and behavior segmentation in a single end-to-end model that leverages learned patterns of animal motion to infer the most plausible pose trajectory from noisy or missing data.

Keypoint-MoSeq is somewhat resilient to noise, but it will perform best with clean keypoint data that capture most parts of the body. Although directly modeling the raw pixel intensities of depth^16^ or 2D video^41^ provides the most detailed access to spontaneous behavior, technical challenges like reflections, occlusions and variation in perspective and illumination remain a challenge in those settings. The development of keypoint-MoSeq—together with advances in markerless pose tracking—should enable MoSeq to be used in a variety of these adversarial circumstances, such as when animals are obstructed from a single axis of view, when multiple animals are interacting simultaneously, when the environment changes dynamically and when animals wear elaborate headgear.

Compared to keypoint-MoSeq, the alternative methods for unsupervised behavior segmentation that we tested (B-SOiD^12^, MotionMapper^17^ and VAME^13^) tend to emit shorter behavior motifs that often start or stop in the middle of what humans might identify as a behavioral module or motif (for example, a rear). Our analysis suggests two possible reasons for this difference. First, unlike alternative methods, MoSeq can discretize behavior at a particular user-defined timescale and, therefore, is better able to identify clear boundaries between behavioral elements that respect the natural rhythmicity in movements associated with syllables, sniffs or steps. The resulting parsimony prevents over-fractionation of individual behaviors. Second, the hierarchical structure of keypoint-MoSeq’s underlying generative model means it can detect noise in keypoint trajectories and distinguish this noise from actual behavior without smoothing away meaningful behavioral transitions.

That said, we stress that there is no one best approach for behavioral analysis, as all methods involve trade-offs^42,43^. For example, keypoint-MoSeq does not yield a single fixed description of behavior, since its output is probabilistic. In principle, one could summarize this uncertainty in the form of a posterior distribution. Because proper posterior estimation is impractical using our current fitting procedure, we have defined an alternative approach whereby users generate an ensemble of candidate model fits and identify a consensus model for downstream analysis. Users wishing to better quantify model uncertainty can also apply subsequent analyses to the full ensemble of models. Keypoint-MoSeq is also limited to describing behavior at a single timescale. Although users may vary this timescale across a broad range, keypoint-MoSeq cannot simultaneously analyze behavior across multiple timescales or explicitly represent the hierarchical nesting of behavior motifs. Finally, because keypoint-MoSeq learns the identity of syllables from the data itself, it may miss especially rare behavioral events that could otherwise be captured using supervised methods.

To facilitate the adoption of keypoint-MoSeq, we built a website (http://www.moseq4all.org/) that includes free access to the code for academics as well as extensive documentation and guidance for implementation. As demonstrated here, the model underlying MoSeq is modular and thus accessible to extensions and modifications that can increase its alignment to behavioral data. For example, a time-warped version of MoSeq was recently reported that incorporates a term to explicitly model variation in movement vigor^19^. We anticipate that the application of keypoint-MoSeq to a wide variety of experimental datasets will both yield important information about the strengths and failure modes of model-based methods for behavioral classification, and prompt continued innovation.

## Methods

### Ethical compliance

All experimental procedures were approved by the Harvard Medical School Institutional Animal Care and Use Committee (protocol number 04930) and were performed in compliance with the ethical regulations of Harvard University as well as the Guide for Animal Care and Use of Laboratory Animals.

### Animal care and behavioral experiments

Unless otherwise noted, behavioral recordings were performed on 8–16-week-old C57/BL6 mice (The Jackson Laboratory stock no. 000664). Mice were transferred to our colony at 6–8 weeks of age and housed in a reverse 12-h light/12-h dark cycle. We single-housed mice after stereotactic surgery and group-housed them otherwise. On recording days, mice were brought to the laboratory, habituated in darkness for at least 20 min, and then placed in the behavioral arena for 30–60 min. We recorded 6 male mice for 10 sessions (6 h) in the initial round of open field recordings; 5 male mice for 52 sessions (50 h) during the accelerometry recordings; 16 male mice for 16 sessions (8 h) during the environmental enrichment experiment; and 5 male mice for 9 sessions (6 h) during the thermistor recordings. The dopamine photometry recordings were obtained from a recent study^22^. They include 6 C57/BL6 mice and 8 DAT-IRES-cre (The Jackson Laboratory stock no. 006660) mice of both sexes, recorded for 378 sessions. Of these, we selected a random subset of 95 sessions (~50 h) for benchmarking keypoint-MoSeq.

### Stereotactic surgery procedures

For all stereotactic surgeries, mice were anesthetized using 1–2% isoflurane in oxygen, at a flow rate of 1 l min^−1^ for the duration of the procedure. Anteroposterior (AP) and mediolateral (ML) coordinates (in millimeters) were zeroed relative to bregma, and the dorsoventral (DV) coordinate was zeroed relative to the pial surface. All mice were monitored daily for 4 days following surgery and were allowed to recover for at least 1 week. Mice were then habituated to handling and brief head-fixation before beginning recordings.

For dopamine recordings, 400 nl of AAV5.CAG.dLight1.1 (Addgene, 111067; titer: 4.85 × 10^12^) was injected at a 1:2 dilution into the DLS (AP 0.260; ML 2.550; DV −2.40), and a single 200-μm-diameter, 0.37–0.57-NA fiber cannula was implanted 200 μm above the injection site (see ref. ^22^ for additional details).

For accelerometry recordings, we surgically attached a Mill-Max connector (DigiKey, ED8450-ND) and head bar to the skull and secured it with dental cement (Metabond). A nine-degree-of-freedom absolute orientation IMU (Bosch, BN0055) was mounted on the Mill-Max connector using a custom printed circuit board (PCB) with a net weight below 1 g.

For thermistor surgeries, we adapted a protocol previously described^37^. We first prepared the implant (GAG22K7MCD419, TE Connectivity) by stripping the leads and soldering them to two male Mill-Max pins (0.05-inch pitch, 851-93-050-10-001000). The pins and their solder joins were then entirely covered in Prime-Dent light-curable cement, and cured for 10–20 s, to ensure the longevity and stability of the electrical connection. Each implant was tested by touching two leads of a multimeter (set to measure resistance) to the female side of the Mill-Max, breathing gently on the thermistor, and checking for a resistance drop of roughly 20 kΩ to 18 kΩ.

To implant the thermistor, a midline incision was made from ~1 mm behind lambda to ~1 mm anterior to the nasal suture, and the skull cleaned and lightly scored. A craniotomy was made just anterior to the nasal suture (well posterior to the position originally reported^37^), large enough for the thermistor to fit fully inside. The thermistor was fully inserted along the AP axis so that it lay flat in the horizontal plane inside the nasal cavity. The craniotomy was then sealed with KwikSil, and the thermistor wire was secured to the skull 1–2 mm posterior to the craniotomy with cyanoacrylate glue (Loctite 454). Then dental cement (Metabond) was used to attach the Mill-Max connector in an upright position between bregma and lambda, and a head bar was cemented to the skull at lambda.

### Microsoft Azure recording setup

For the initial set of open field recordings (Figs. 1, 2, 3a–g and 5g–l), mice were recorded in a square arena with transparent floor and walls (30 cm length and width). Microsoft Azure Kinect cameras captured simultaneous depth and near-IR video at 30 Hz. Six cameras were used in total: one above, one below and four side cameras at right angles at the same height as the mouse.

### Accelerometry recordings

For the accelerometry recordings, we used a single Microsoft Azure Kinect camera placed above the mouse, and an arena with transparent floor and opaque circular walls (45-cm diameter). Data were transferred from the IMU using a lightweight tether attached to a custom-built active commutator. The IMU was connected to a Teensy microcontroller, which was programmed using the Adafruit BNO055 library with default settings (sample rate: 100 Hz, units: m/s^2^). To synchronize the IMU measurements and video recordings, we used an array of near-IR LEDs to display a rapid sequence of random 4-bit codes that updated throughout the recording. The code sequence was later extracted from the behavioral videos and used to fit a piecewise linear model between timestamps from the videos and timestamps from the IMU.

### Thermistor recordings

To record mouse respiration and movement at high frame rates, we built a multi-camera recording arena using six Basler ace acA1300-200um Monochrome USB 3.0 Cameras (Edmund Optics, 33-978) that recorded from above, from below and four side views. The cameras were triggered at 120 Hz using an Arduino. Video compression was performed in real time on a GPU using a custom library (https://github.com/calebweinreb/multicamera_acquisition/). Mice were recorded in an open-top glass cube and illuminated with 32 near-IR high-power LED stars (LEDSupply, CREEXPE-FRD-3). To avoid reflections and saturations effects, the bottom camera was triggered slightly out of phase with the top cameras, and the LEDs were split into two groups: one group below the arena that turned on during the bottom camera’s exposure, and one group above the arena that turned on during the top and side cameras’ exposure.

To record the thermistor signal, we designed a custom PCB that used an op-amp (INA330AIDGST, Texas Instruments) to transform the thermistor’s resistance fluctuations into voltages, and another circuit element to keep the voltage within the 0–3.3 V range. The PCB was connected to an Arduino (separate from the one controlling the cameras) that recorded the output. The PCB parts list, schematic and microcontroller code are available upon reasonable request to the laboratory of S.R.D.

Before behavioral recording sessions with the thermistor, mice were briefly head-fixed, and a cable with a custom headstage was inserted into the head-mounted Mill-Max adaptor. The cable was commutated with an assisted electric commutator from Doric Lenses and connected to the input of the op-amp on the custom PCB. To synchronize the thermistor and video data, we piped a copy of the camera trigger signal from the camera-Arduino to the thermistor-Arduino and recorded this signal alongside the thermistor output.

### Environmental enrichment recordings

To test the effects of environmental enrichment on behavior, we built an arena for overhead video recording of an open-topped home cage. The home cage was surrounded on each side by a 16-inch vertical barrier, illuminated from above by three near-IR LED starts (LEDSupply, CREEXPE-FRD-3) and recorded with a Basler ace acA1300-200um Monochrome USB 3.0 Camera (Edmund Optics 33-978). For half the recordings, the cage was filled with bedding, nesting material, chew sticks and a transparent, dome-shaped hut. For the other half, the cage was completely empty (except for the mouse).

### Software

The following publicly available software packages were used for analysis: Python (version 3.8), NumPy (version 1.24.3), Scikit-learn (version 1.2.2), PyTorch (version 1.9), Jax (version 0.3.22), SciPy (version 1.10.1), Matplotlib (version 3.7.1), Statsmodels (version 0.13.5), Motionmapperpy (version 1.0), DeepLabCut (version 2.2.1), SLEAP (version 1.2.3), B-SOiD (version 1.5.1), VAME (version 1.1), GIMBAL (version 0.0.1), HRNet (unversioned), LightningPose (version 0.0.4) and segmentation_models_pytorch (version 0.3.3).

### Statistics

All reported *P* values for comparisons between distributions were derived from Mann–Whitney *U* tests unless stated otherwise. In all comparisons to ‘shuffle’, the shuffle represents a cyclic permutation of the data.

### Processing depth videos

Applying MoSeq to depth videos involves: (1) mouse tracking and background subtraction; (2) egocentric alignment and cropping; (3) PCA; and (4) probabilistic modeling. We applied steps 2–4 as described in the MoSeq2 pipeline^25^. For step 1, we trained a convolutional neural network (CNN) with a Unet++^44^ architecture to segment the mouse from background using ~5,000 hand-labeled frames as training data.

### Keypoint tracking for Microsoft Azure IR recordings

We used CNNs with an HRNet^45^ architecture (https://github.com/stefanopini/simple-HRNet/) with a final stride of two for pose tracking. The networks were trained on ~1,000 hand-labeled frames each for the overhead, below-floor and side-view Microsoft Azure cameras. Frame labeling was crowdsourced through a commercial service (Scale AI). The crowdsourced labels were comparable to those from experts in our laboratory (Extended Data Fig. 1d). For the overhead camera, we tracked two ears and six points along the dorsal midline (tail base, lumbar spine, thoracic spine, cervical spine, head and nose). For the below-floor camera, we tracked the tip of each forepaw, the tip and base of each hind paw, and four points along the ventral midline (tail base, genitals, abdomen and nose). For the side cameras, we tracked the same eight points as for the overhead camera and included the six limb points that were used for the below-floor camera (14 total). We trained a separate CNN for each camera angle. Target activations were formed by centering a Gaussian with a 10-pixel (px) standard deviation on each keypoint. We used the location of the maximum pixel in each output channel of the neural network to determine keypoint coordinates and used the value at that pixel to set the confidence score. The resulting mean absolute error (MAE) between network detections and manual annotations was 2.9 px for the training data and 3.2 px for held-out data. We also trained DeepLabCut and SLEAP models on the overhead-camera and below-floor-camera datasets. For DeepLabCut, we used version 2.2.1, setting the architecture to resnet50 architecture and the ‘pos_dist_thresh’ parameter to 10, resulting in train and test MAEs of 3.4 px and 3.8 px, respectively. For SLEAP, we used version 1.2.3 with the baseline_large_rf.single.json configuration, resulting in train and test MAEs of 3.5 px and 4.7 px. For Lightning Pose^40^, we used version 0.0.4 and default parameters with ‘pca_singleview’ and ‘temporal’ loss terms.

### Keypoint tracking for thermistor recordings

We trained separate keypoint detection networks for the Basler camera arena (used for the thermistor recordings). CNNs with an HRNet architecture were trained on ~1,000 hand-labeled frames each for the overhead and below-floor cameras and ~3,000 hand-labeled frames for the side-view cameras. The same keypoints were used as the ones for the Microsoft Azure dataset.

### 3D pose inference

Using 2D keypoint detections from six cameras, 3D keypoint coordinates were triangulated and then refined using GIMBAL, a model-based approach that leverages anatomical constraints and motion continuity^29^. To fit GIMBAL, we computed initial 3D keypoint estimates using robust triangulation (that is, by taking the median across all camera pairs, as in 3D-DeepLabCut^46^) and then filtered to remove outliers using the EllipticEnvelope method from sklearn; we then fit the skeletal parameters and directional priors for GIMBAL using expectation maximization with 50 pose states. Finally, we applied the fitted GIMBAL model to each recording, using the following parameters for all keypoints: obs_outlier_variance = 1e6, obs_inlier_variance = 10, pos_dt_variance = 10. The latter parameters were chosen based on the accuracy of the resulting 3D keypoint estimates, as assessed from visual inspection. Camera calibration and initial triangulation were performed using a custom library (https://github.com/calebweinreb/multicam-calibration/tree/main/multicam_calibration/).

### Keypoint change score

We defined the keypoint ‘change score’ as the total velocity of keypoints after egocentric alignment. The goal of the change score is to highlight sudden shifts in pose. It was calculated by: (1) transforming keypoints into egocentric coordinates; (2) smoothing the transformed coordinates with Gaussian kernel (sigma = 1 frame); (3) calculating total change in coordinates across each frame; and (4) *z*-scoring. Formally, the score can be defined as:

$${\rm{Change}}\,{\rm{score}}\left(t\right)={z\,{\mathrm{score}}}(\left|\;{y}_{{t}}-{y}_{{t-1}}\right|)$$

where *y*_*t*_ are the keypoint coordinates after Gaussian smoothing.

### Spectral analysis of keypoint jitter

To analyze keypoint jitter, we quantified the magnitude of fluctuations across a range of frequencies by computing a spectrogram for each keypoint along each coordinate axis. Spectrograms were computed using the python function scipy.signal.spectrogram with nperseg = 128 and noverlap = 124. The spectrograms were then combined through averaging: each keypoint was assigned a spectrogram by averaging over the two coordinate axes, and the entire animal was assigned a spectrogram by averaging over all keypoints.

We used the keypoint-specific spectrograms to calculate cross-correlations with −log_10_ (neural network detection confidence), as well as the ‘error magnitude’ (Fig. 1g). Error magnitude was defined as the distance between the detected 2D location of a keypoint (based on a single camera angle) and a re-projection of its 3D position (based on consensus across six camera angles; see ‘3D pose inference’ above). We also computed the cross-correlation between nose and tail-base fluctuations at each frequency, as measured by the overhead and below-floor cameras, respectively. Finally, we averaged spectral power across keypoints to compute the cross-correlation with model transition probabilities (Fig. 1g). The model transition probabilities were defined for each frame as the fraction of *N* = 20 model fits in which a transition occurred on that frame. Formally, if *z*^(*i*)^ denotes the syllable sequence learned by model fit *i*, then the transition probability at time *t* is calculated as

$$\frac{1}{N}\mathop{\sum }\limits_{i=1}^{N}\delta \left({z}_{{t}}^{\left({i}\right)}\ne {z}_{{t-t}}^{\left({i}\right)}\right)$$

### Applying keypoint-MoSeq

Datasets were modeled separately and multiple models with different random seeds were fit for each dataset (see Supplementary Table 1 for number of fits per dataset).

Modeling consisted of two phases: (1) fitting an AR-HMM to a fixed pose trajectory derived from PCA of egocentric-aligned keypoints; and (2) fitting a full keypoint-MoSeq model initialized from the AR-HMM. References in the text to ‘MoSeq applied to keypoints’ or ‘MoSeq (keypoints)’, for example, in Figs. 1 and 2, refer to output of step 1. Both steps are described below, followed by a detailed description of the model and inference algorithm in the ‘mathematical notation’ section. In all cases, we excluded rare states (frequency < 0.5%) from downstream analysis. We have made the code available as a user-friendly package via https://keypoint-moseq.readthedocs.io/en/latest/. With a consumer GPU, keypoint-MoSeq requires 30–60 min of computation time to model 5 h of data. The computation time scales linearly with dataset size.

### Fitting an initial AR-HMM

We first modified the keypoint coordinates, defining keypoints with confidence below 0.5 as missing data and in imputing their values via linear interpolation, and then augmenting all coordinates with a small amount of random noise; the noise values were uniformly sampled from the interval [−0.1, 0.1] and helped prevent degeneracy during model fitting. Importantly, these preprocessing steps were only applied during AR-HMM fitting—the original coordinates were used when fitting the full keypoint-MoSeq model.

Next, we centered the coordinates on each frame, aligned them using the tail–nose angle, and then transformed them using PCA with whitening. The number of principal components (PCs) was chosen for each dataset as the minimum required to explain 90% of total variance. This resulted in four PCs for the overhead-camera 2D datasets, six PCs for the below-floor camera 2D datasets and six PCs for the 3D dataset.

We then used Gibbs sampling to infer the states and parameters of an AR-HMM, including the state sequence *z*, the autoregressive parameters *A*, *b* and *Q*, and the transition parameters *π* and *β*. The hyperparameters for this step, listed in ‘mathematical notation’ below, were generally identical to those in the original depth MoSeq model. The one exception was the stickiness hyperparameter *κ*, which we adjusted separately for each dataset to ensure a median state duration of 400 ms.

### Fitting a full keypoint-MoSeq model

We next fit the full set of variables for keypoint-MoSeq, which include the AR-HMM variables mentioned above, as well as the location *v* and heading *h*, latent pose trajectory *x*, per-keypoint noise level *σ*^2^ and per-frame/per-keypoint noise scale *s*. Fitting was performed using Gibbs sampling for 500 iterations, at which point the log joint probability appeared to have stabilized.

The hyperparameters for this step are enumerated in ‘mathematical notation’. In general, we used the same hyperparameter values across datasets. The two exceptions were the stickiness hyperparameter *κ*, which again had to be adjusted to maintain a median state duration of 400 ms, and *s*_0_, which determines a prior on the noise scale. Because low-confidence keypoint detections often have high error, we set *s*_0_ using a logistic curve that transitions between a high-noise regime (*s*_0_ = 100) for detections with low confidence and a low-noise regime (*s*_0_ = 1) for detections with high confidence:

$${s}_{0}=1+100{\left(1+{\rm{e}}^{20\left({\rm{confidence}}-0.4\right)}\right)}^{-1}$$

The *κ* value used for each dataset is reported in Supplementary Table 2.

### Trajectory plots

To visualize the modal trajectory associated with each syllable (Fig. 2e), we (1) computed the full set of trajectories for all instances of all syllables, (2) used a local density criterion to identify a single representative instance of each syllable and (3) computed a final trajectory using the nearest neighbors of the representative trajectory.

### Computing the trajectory of individual syllable instances

Let *y*_*t*_, *v*_*t*_ and *h*_*t*_ denote the keypoint coordinates, centroid and heading of the mouse at time *t*, and let *F*(*v*, *h*; *y*) denote the rigid transformation that egocentrically aligns *y* using centroid *v* and heading *h*. Given a syllable instance with onset time *T*, we computed the corresponding trajectory *X*_*T*_ by centering and aligning the sequence of poses $({y}_{{T-5}},\ldots ,{y}_{{T+15}})$ using the centroid and heading on time *T*. In other words

$${X}_{{T}}=\left[F\left({v}_{{T}},{h}_{{T}}{\rm{;}}{y}_{{T-5}}\right),\ldots ,F\left({v}_{{T}},{h}_{{T}}{\rm{;}}{y}_{{T+15}}\right)\right]$$

### Identifying a representative instance of each syllable

The collection of trajectories computed above can be thought of as a set of points in a high dimensional trajectory space (for *K* keypoints in 2D, this space would have dimension 40*K*). Each point has a syllable label, and the segregation of these labels in the trajectory space represents the kinematic differences between syllables. To capture these differences, we computed a local probability density function for each syllable, and a global density function across all syllables. We then selected a representative trajectory *X* for each syllable by maximizing the ratio:

$$\frac{{\rm{Local}}\, {\rm{density}}(X)}{{\rm{Global}}\, {\rm{density}}(X)}$$

The density functions were computed as the mean distance from each point to its 50 nearest neighbors. For the global density, the nearest neighbors were selected from among all instances of all syllables. For the local densities, the nearest neighbors were selected from among instances of the target syllable.

### Computing final trajectories for each syllable

For each syllable and its representative trajectory *X*, we identified the 50 nearest neighbors of *X* from among other instances of the same syllable and then computed a final trajectory as the mean across these nearest neighbors. The trajectory plots in Fig. 2e consist of ten evenly-space poses along this trajectory, that is, the poses at times $T-5,{T}-3,\ldots ,T+13$.

### Testing robustness to missing data

To test the ability of keypoint-MoSeq to infer syllables and sequences in the face of missing data, we artificially ablated random subsets of keypoints at randomly timed intervals and then modeled the ablated data (Extended Data Fig. 2c–f). The ablation intervals began on every 10th second of the recording and lasted between 33 ms and 3 s (uniformly at random). For each interval, anywhere between 1 and 8 keypoints were selected (uniformly at random). Ablation entailed (1) erasing the keypoint coordinates and then filling the gap by linear interpolation; (2) setting the corresponding confidence values to 0. We then applied keypoint-MoSeq 20 times with different random seeds, using a single, fixed set of parameters derived previously from standard model fitting on the unablated dataset. Fixing the parameters ensured that syllable labels would be comparable across repeated model fits.

### Cross-syllable likelihoods

We defined each cross-syllable likelihood as the probability (on average) that instances of one syllable could have arisen based on the dynamics of another syllable. The probabilities were computed based on the discrete latent states *z*_*t*_, continuous latent states *x*_*t*_ and autoregressive parameters *A*, *b* and *Q* output by keypoint-MoSeq. The instances *I*(*n*) of syllable *n* were defined as the set of all sequences $({t}_{\rm{s}},\ldots ,{t}_{\rm{e}})$ of consecutive timepoints such that *z*_*t*_ = *n* for all ${t}_{\rm{s}}\le t\le {t}_{\rm{e}}$ and ${z}_{{t}_{\rm{s}}-1}\ne n\ne {z}_{{t}_{\rm{e}}+1}$. For each such instance, one can calculate the probability $P\left({x}_{{t}_{\rm{s}}},\ldots ,{x}_{{t}_{\rm{e}}}\right|{A}_{{m}},{b}_{{m}},{Q}_{{m}})$ that the corresponding sequence of latent states arose from the autoregressive dynamics of syllable *m*. The cross-syllable likelihood *C*_*nm*_ is defined in terms of these probabilities as

$${C}_{{nm}}=\frac{1}{\left|I(n)\right|}\sum _{\left({{t}}_{\rm{s}},\ldots ,{{t}}_{\rm{e}}\right)\in {\rm{I}}({{n}})}\frac{\left({x}_{{t}_{\rm{s}}},\ldots ,{x}_{{t}_{\rm{e}}}\right|{A}_{{m}},{b}_{{m}},{Q}_{{m}})}{\left({x}_{{t}_{\rm{s}}},\ldots ,{x}_{{t}_{\rm{e}}}\right|{A}_{{n}},{b}_{{n}},{Q}_{{n}})}$$

### Generating synthetic keypoint data

To generate the synthetic keypoint trajectories used for Extended Data Fig. 2h, we fit a linear dynamical system (LDS) to egocentrically aligned keypoint trajectories and then sampled randomly generated outputs from the fitted model. The LDS was identical to the model underlying keypoint-MoSeq (see ‘mathematical notation’), except that it only had one discrete state, lacked centroid and heading variables and allowed separate noise terms for the *x* and *y* coordinates of each keypoint.

### Expected marginal likelihood score

Because keypoint-MoSeq can at best produce point estimates of the model parameters—which will differ from run to run—users typically run the model several times and then rank the resulting fits. For ranking model fits, we defined a custom metric called the expected marginal likelihood score. The score evaluates a given set of autoregressive parameters (*A*, *b*, *Q*) by the expected value of the marginal log likelihood: ${E}_{{x} \sim P\left(x|\;y\right)}\log P\left(x|A,b,Q\right)$. In practice, given an ensemble of pose trajectories *x*^(*i*)^ and parameters ${\theta }^{(i)}=(A,b,Q)$ derived from *N* separate MCMC chains, the scores are computed as:

$${\rm{Score}}\left({\theta }^{\left({i}\right)}\right)=\frac{1}{1-N}\sum _{{j\ne i}}\log P\left({{x}^{\,\left({j}\,\right)}{\rm{|}}\theta }^{(i)}\right)$$

The scores shown in Extended Data Fig. 3j–m were computed using an ensemble of *N* = 20 chains. We chose this custom score instead of a more standard metric (such as held-out likelihood) because computing the latter is intractable for the keypoint-MoSeq model.

### Environmental enrichment analysis

We fit a single keypoint-MoSeq model to the environmental enrichment dataset, which included recordings in an enriched home cage and control recordings in an empty cage. The transition graph (Extended Data Fig. 8b) was generated with keypoint-MoSeq’s analysis pipeline (https://keypoint-moseq.readthedocs.io/en/latest/analysis.html#syllable-transition-graph/) using node positions from a force directed layout. Detection of differentially used syllables was also performed using the analysis pipeline, which applies a Kruskal–Wallis test for significant differences in the per-session frequency of each syllable (https://keypoint-moseq.readthedocs.io/en/latest/analysis.html#compare-between-groups/). Syllables were clustered into three groups by applying community detection (networkx.community.louvain_communities) to a complete graph where nodes are syllables and edges were weighted by the bigram probabilities ${b}_{ij}=P({z}_{t}=i,\,{z}_{t+1}=j)$).

### Applying published methods for behavior analysis

We applied B-SOiD, VAME and MotionMapper using default parameters, except for the parameter scans in Extended Data Fig. 5 (see Supplementary Table 3 for a summary for all parameter choices). In general, we were unable to uniformly improve the performance of any method by deviating from these default parameters. For example, switching VAME’s state-partition method from hidden Markov model (HMM) to *k*-means led to higher change score alignment (Extended Data Fig. 5a) but caused a decrease in alignment to supervised behavior labels (Fig. 5e,f shows performance under an HMM; performance under *k*-means is not shown). Our application of each method is described in detail below.

B-SOiD is an automated pipeline for behavioral clustering that: (1) preprocesses keypoint trajectories to generate pose and movement features; (2) performs dimensionality reduction on a subset of frames using uniform manifold approximation and projection; (3) clusters points in the uniform manifold approximation and projection space; and (4) uses a classifier to extend the clustering to all frames^12^. We fit B-SOiD separately for each dataset. In each case, steps 2–4 were performed multiple times with different random seeds (see Supplementary Table 1 for number of fits per dataset), and the pipeline was applied with standard parameters; 50,000 randomly sampled frames were used for dimensionality reduction and clustering, and the min_cluster_size range was set to 0.5–1%. Because B-SOiD uses a hardcoded window of 100 ms to calculate pose and movement features, we reran the pipeline with falsely inflated frame rates for the window-size scan in Extended Data Fig. 5a. In all analyses involving B-SOiD, rare states (frequency < 0.5%) were excluded from the analysis.

VAME is a pipeline for behavioral clustering that: (1) preprocesses keypoint trajectories and transforms them into egocentric coordinates; (2) fits a recurrent neural network; (3) clusters the latent code of the recurrent neural network^13^. We applied these steps separately to each dataset, in each case running step 3 multiple times with different random seeds (see Supplementary Table 1 for number of fits per dataset). For step 1, we used the same parameters as in keypoint-MoSeq—egocentric alignment was performed along the tail–nose axis, and we set the pose_confidence threshold to 0.5. For step 2, we set time_window = 30 and zdims = 30 for all datasets, except for the zdim-scan in Extended Data Fig. 5a. VAME provides two different options for step 3: fitting an HMM (default) or applying *k*-means (alternative). We fit an HMM for all datasets and additionally applied *k*-means to the initial open dataset. In general, we approximately matched the number of states/clusters in VAME to the number identified by keypoint-MoSeq, except when scanning over state number in Extended Data Fig. 5a. In all analyses involving VAME, rare states (frequency < 0.5%) were excluded from analysis.

MotionMapper performs unsupervised behavioral segmentation by: (1) applying a wavelet transform to preprocessed pose data; (2) nonlinearly embedding the transformed data in 2D; and (3) clustering the 2D data with a watershed transform^17^. We applied these steps separately to each dataset, in each case running steps 2–3 multiple times with different random seeds (see Supplementary Table 1 for number of fits per dataset). There are several published implementations of MotionMapper, which perform essentially the same set of transformations but differ in programming language. We obtained similar results from a recent Python implementation from the Berman laboratory (https://github.com/bermanlabemory/motionmapperpy/) and a published MATLAB implementation^30^. All results in the paper are from the Python implementation, which we applied as follows. Data were first egocentrically aligned along the tail–nose axis and then projected into eight dimensions using PCA. Ten log-spaced frequencies between 0.25 Hz and 15 Hz were used for the wavelet transform, and dimensionality reduction was performed using *t*-distributed stochastic neighbor embedding. The threshold for watershedding was chosen to produce at least 25 clusters, consistent with keypoint-MoSeq for the overhead-camera data. Rare states (frequency < 0.5%) were excluded from analysis. For the parameter scan in Extended Data Fig. 5a, we varied each of these parameters while holding the others fixed, including the threshold for watershedding, the number of initial PCA dimensions, and the frequency range of wavelet analysis. We also repeated a subset of these analyses using an alternative autoencoder-based dimensionality reduction approach, as described in the motionmapperpy tutorial (https://github.com/bermanlabemory/motionmapperpy/blob/master/demo/motionmapperpy_mouse_demo.ipynb/).

### Predicting kinematics from state sequences

We trained decoding models based on spline regression to predict kinematic parameters (height, velocity and turn speed) from state sequences output by keypoint-MoSeq and other behavior segmentation methods (Fig. 3e and Extended Data Fig. 5c). Let *z*_*t*_ represent an unsupervised behavioral state sequence and let *B* denote a spline basis, where *B*_*t*,*i*_ is the value of spline *i* and frame *t*. We generated such a basis using the ‘bs’ function from the Python package ‘patsy’, passing in six log-spaced knot locations (1.0, 2.0, 3.9, 7.7, 15.2 and 30.0) and obtaining basis values over a 300-frame interval. This resulted in a 300-by-5 basis matrix *B*. The spline basis and state sequence were combined to form a 5*N*-dimensional design matrix, where *N* is the number of distinct behavioral states. Specifically, for each instance $({t}_{\rm{s}},\ldots ,{t}_{\rm{e}})$ of state *n* (see ‘Cross-syllable likelihoods’ for a definition of state instances), we inserted the first ${t}_{\rm{e}}-{t}_{\rm{s}}$ frames of *B* into dimensions $5n,\ldots ,5n+5$ of the design matrix, aligning the first frame of *B* to frame *t*_s_ in the design matrix. Kinematic features were regressed against the design matrix using Ridge regression from scikit-learn and fivefold cross-validation. We used a range of values from 10^−3^ to 10^3^ for the regularization parameter *α* and reported the results with greatest accuracy.

### Rearing analysis

To compare the dynamics of rear-associated states across methods, we systematically identified all instances of rearing in our initial open field dataset. During a stereotypical rear, mice briefly stood on their hind legs and extended their head upwards, leading to a transient increase in height from its modal value of 3–5 cm to a peak of 7–10 cm. Rears were typically brief, with mice exiting and then returning to a prone position within a few seconds. We encoded these features using the following criteria. First, rear onsets were defined as increases in height from below 5 cm to above 7 cm that occurred within the span of a second, with onset formally defined as the first frame where the height exceeded 5 cm. Next, rear offsets were defined as decreases in height from above 7 cm to below 5 cm that occurred within the span of a second, with offset formally defined as the first frame where the height fell below 7 cm. Finally, we defined complete rears as onset–offset pairs defining an interval with length between 0.5 s and 2 s. Height was determined from the distribution of depth values in cropped, aligned and background-segmented videos. Specifically, we used the 98th percentile of the distribution in each frame.

### Accelerometry processing

From the IMU, we obtained absolute rotations *r*_y_, *r*_p_ and *r*_r_ (yaw, pitch and roll) and accelerations *a*_*x*_, *a*_*y*_ and *a*_*z*_ (dorsal/ventral, posterior/anterior and left/right). To control for subtle variations in implant geometry and chip calibration, we centered the distribution of sensor readings for each variable on each session. We defined total acceleration as the norm of the three acceleration components:

$$\left|a\right|=\sqrt{{a}_{{x}}^{2}+{a}_{{y}}^{2}+{a}_{{z}}^{2}}$$

Similarly, we defined total angular velocity as the norm |*ω*| of rotation derivative:

$$\omega =\left(\frac{{\rm{d}}{r}_{{y}}}{{{\rm{d}}t}},\frac{{\rm{d}}{r}_{{p}}}{{{\rm{d}}t}},\frac{{\rm{d}}{r}_{{r}}}{{{\rm{d}}t}}\right)$$

Finally, to calculate jerk, we smoothed the acceleration signal with a 50-ms Gaussian kernel, generating a time series $\widetilde{a}$, and then computed the norm of its derivative:

$${\rm{Jerk}}=\left|\frac{{\rm{d}}\widetilde{a}}{{{\rm{d}}t}}\right|$$

### Aligning dopamine fluctuations to behavior states

For a detailed description of photometry data acquisition and preprocessing, see ref. ^22^. Briefly, photometry signals were: (1) normalized using Δ*F/F*_0_ with a 5-s window; (2) adjusted against a reference to remove motion artifacts and other non-ligand-associated fluctuations; (3) *z*-scored using a 20-s sliding window; and (4) temporally aligned to the 30-Hz behavioral videos.

Given a set of state onsets (either for a single state or across all states), we computed the onset-aligned dopamine trace by averaging the dopamine signal across onset-centered windows. From the resulting traces, each of which can be denoted as a time series of dopamine signal values (${d}_{{-T}},\ldots ,{d}_{{T}}$), we defined the total fluctuation size (Fig. 4d) and temporal asymmetry (Fig. 4e) as

$$\begin{array}{l}{\rm{Temporal}}\,{\rm{asymmetry}}=\frac{1}{15}\mathop{\sum }\limits_{t=0}^{15}{d}_{\rm{t}}-\frac{1}{15}\mathop{\sum }\limits_{t=-15}^{0}{d}_{{t}} \\{\rm{Total}}\, {\rm{fluctuation}}\,{\rm{size}}=\mathop{\sum}\limits_{t=-15}^{15}\left|{d}_{{t}}\right|\end{array}$$

A third metric—the average dopamine during each state (Extended Data Fig. 7b)—was defined simply as the mean of the dopamine signal across all frames bearing that state label. For each metric, shuffle distributions were generated by repeating the calculation with a temporally reversed copy of the dopamine times series.

### Supervised behavior benchmark

Videos and behavioral annotations for the supervised open field behavior benchmark (Fig. 5a–c) were obtained from ref. ^31^. The dataset contains 20 videos that are each 10–20-min long. Each video includes frame-by-frame annotations of five possible behaviors: locomote, rear, face groom, body groom and defecate. We excluded ‘defecate’ from the analysis because it was extremely rare (<0.1% of frames).

For pose tracking, we used DLC’s SuperAnimal inference API that performs inference on videos without the need to annotate poses in those videos^47^. Specifically, we used SuperAnimal-TopViewMouse that applies DLCRNet-50 as the pose estimation model. Keypoint detections were obtained using DeepLabCut’s API function deeplabcut.video_inference_superanimal. The API function uses a pretrained model called SuperAnimal-TopViewMouse and performs video adaptation that applies multi-resolution ensemble (that is, the image height resized to 400, 500 and 600 with a fixed aspect ratio) and rapid self-training (model trained on zero shot predictions with confidence above 0.1) for 1,000 iterations to counter domain shift and reduce jittering predictions.

Keypoint coordinates and behavioral annotations for the supervised social behavior benchmark (Fig. 5d–f) were obtained from the CalMS21 dataset^32^ (task1). The dataset contains 70 videos of resident–intruder interactions with frame-by-frame annotations of four possible behaviors: attack, investigate, mount or other. All unsupervised behavior segmentation methods were fitted to 2D keypoint data for the resident mouse.

We used four metrics^13^ to compare supervised annotations and unsupervised states from each method. These included NMI, homogeneity, adjusted rand score and purity. All metrics besides purity were computed using the Python library scikit-learn (that is, with the function normalized_mutual_info_score, homogeneity_score, adjusted_rand_score). The purity score was defined as in ref. ^13^.

### Thermistor signal processing

During respiration, the movement of air through a mouse’s nasal cavity generates fluctuations in temperature that can be detected by a thermistor; temperature decreases during inhalations (because the mouse is warmer than the air around it) and rises between inhalations. Below we refer to the between-inhalation intervals as ‘exhales’ but note that they may also contain pauses in respiration—pauses and exhales likely cannot be distinguished because warming of the thermistor occurs whether or not air is flowing.

To segment inhales and exhales using the thermistor signal, we first applied a 60-Hz notch filter (scipy.signal.iirnotch, *q* = 10) and a low-pass filter (scipy.signal.butter, order = 3, cutoff = 40 Hz, analog = false) to the raw signal, and then used a median filter to subtract the slow DC offset component of the signal. We then performed peak detection using scipy.signal.find_peaks (minimium inter-peak distance of 50 ms, minimum and maximum widths of 10 ms and 1,500 ms, respectively). To distinguish true peaks (inhalation onsets) from spurious peaks (noise), we varied the minimum prominence parameter from 10^−4^ to 1 while keeping other parameters fixed, and then used the value at which the number of peaks stabilized. Using the chosen minimum prominence, the signal was then analyzed twice—once at the chosen value, and again with a slightly more permissive minimum prominence (1/8 of the chosen value). Any low-amplitude breaths detected with the more permissive setting that overlapped with periods of breathing between 1 Hz and 6 Hz were added to the detections. This same process was then repeated to find exhale onsets but with the thermistor signal inverted. Finally, inhales and exhales were paired, and any instances of two inhales/exhales in a row were patched by inserting an exhale/inhale at the local extremum between them. Detections were then inspected manually, and any recordings with excessive noise, unusually high breathing rates (>14 Hz), or unusual autocorrelation profiles were removed from further analyses.

### Classifying sniff-aligned syllables

To test whether syllables were significantly sniff aligned, we compared the probability of inhalation in the 50 ms before versus 50 ms after syllable onset. Specifically, for each syllable, we quantified the pre-inhalation versus post-inhalation fraction across all instances of that syllable, and then compared the pre-distribution and post-distribution values using a paired *t*-test. Syllables with *P* < 0.001 were considered significant.

### Fly gait analysis

For the analysis of fly behavior, we used a published dataset of keypoint coordinates^39^, which were derived from behavioral videos originally reported in ref. ^17^. The full dataset contains 1-h recordings (100 fps) of single flies moving freely on a backlit 100-mm-diameter arena. Keypoints were tracked using LEAP (test accuracy ~2.5 px). MotionMapper results (including names for each cluster) were also included in the published dataset. We chose four 1-h sessions (uniformly at random) for analysis with keypoint-MoSeq. All results reported here were derived from this 4-h dataset.

The analysis of syllable probabilities across the stride cycle (Fig. 6i–k) was limited to periods of ‘fast locomotion’, as defined by the MotionMapper labeling (state label 7). To identify the start and end of each stride cycle, we applied PCA to egocentric keypoint coordinates (restricted to fast locomotion frames). We found that the first PC oscillated in a manner reflecting the fly’s gait, and thus smoothed the first PC using a one-frame Gaussian filter and performed peak detection on the smoothed signal. Each inter-peak interval was defined as one stride. Stances and swings (Fig. 6j and Extended Data Fig. 10g) were defined by backward and forward motion of the leg tips, respectively (in egocentric coordinates).

### Mathematical notation

1. *χ*^−2^(*ν*, *τ*^2^) denotes the scaled inverse Chi-squared distribution.
2. $\otimes$ denotes the Kronecker product.
3. *Δ*^*N*^ is the *N*-dimensional simplex.
4. *I*_*N*_ is the *N* × *N* identity matrix.
5. **1**_*N* × *M*_ is the *N* × *M* matrix of ones.
6. $\bf{x}_{{t}_{1}:{t}_{2}}$ denotes the concatenation $\left[\bf{x}_{{t}_{1}},\bf{x}_{{t}_{1}+1},\ldots ,\bf{x}_{{t}_{2}}\right]$ where *t*_1 _< *t*_2_.

### Generative model

Keypoint-MoSeq learns syllables by fitting an SLDS model^48^, which decomposes an animal’s pose trajectory into a sequence of stereotyped dynamical motifs. In general, SLDS models explain time-series observations *y*_1_, …, *y*_*T*_ through a hierarchy of latent states, including continuous states $\bf{x}_{{t}}\in {{\mathbb{R}}}^{{M}}$ that represent the observations *y*_*t*_ in a low-dimensional space, and discrete states *z*_*t*_ ∈ {1, …, *N*} that govern the dynamics of **x**_*t*_ over time. In keypoint-MoSeq, the discrete states correspond to syllables, the continuous states correspond to pose, and the observations are keypoint coordinates. We further adapted SLDS by (1) including a sticky hierarchical Dirichlet prior (HDP); (2) explicitly modeling the animal’s location and heading; and (3) including a robust (heavy-tailed) observation distribution for keypoints. Below we review SLDS models in general and then describe each of the customizations implemented in keypoint-MoSeq.

### SLDSs

The discrete states *z*_*t*_ ∈ {1, …, *N*} are assumed to form a Markov chain, meaning

$${z}_{{t+1}}{\rm{| }}{z}_{{t}}\sim {\rm{Cat}}\left({\pi }_{{z}_{{t}}}\right)$$

where ${\pi }_{i}\in {\Delta }^{N}$ is the probability of transitioning from discrete state *i* to each other state. Conditional on the discrete states *z*_*t*_, the continuous states *x*_*t*_ follow an *L*-order vector autoregressive process with Gaussian noise. This means that the expected value of each *x*_*t*_ is a linear function of the previous *L* states $\bf{x}_{{t-L:t-1}}$, as shown below

$${x}_{{t}}{\rm{| }}{z}_{{t}},{x}_{{t-L:t-1}}{\mathscr{\sim }}{\mathscr{N}}\left({A}_{{z}_{t}}{x}_{{t-L:t-1}}+{b}_{{z}_{{t}}},{Q}_{{z}_{{t}}}\right)$$

where ${A}_{{i}}\in {{\mathbb{R}}}^{{M\times {LM}}}$ is the autoregressive dynamics matrix, $\bf{b}_{{i}}\in {{\mathbb{R}}}^{{M}}$ is the dynamics bias vector, and ${Q}_{{i}}\in {{\mathbb{R}}}^{{M\times M}}$ is the dynamics noise matrix for each discrete state *i* = 1, …, *N*. The dynamics parameters *A*_*i*_, **b**_*i*_ and *Q*_*i*_ have a matrix normal inverse Wishart (MNIW) prior

$$\left[{A}_{{i}}{\rm{| }}\bf{b}_{{i}}\right],{Q}_{{i}}\sim {\rm{MNIW}}\left({\nu }_{0},{S}_{0},{M}_{0},{K}_{0}\right)$$

where *ν*_0_ > M − 1 is the degrees of freedom, ${S}_{0}\in {{\mathbb{R}}}^{{M\times M}}$ is the prior covariance matrix, ${M}_{0}\in {{\mathbb{R}}}^{{M\times \left({LM}+1\right)}}$ is the prior mean dynamics matrix, and ${K}_{0}\in {{\mathbb{R}}}^{\left({{LM}}+1\right)\times \left({{L}M}+1\right)}$ is the prior scale matrix. Finally, in the standard formulation of SLDS (which we modify for keypoint data, as described below), each observation $\bf{y}_{{t}}\in {{\mathbb{R}}}^{{D}}$ is a linear function of **x**_*t*_ plus noise:

$$\bf{y}_{{t}}{\rm{| }}{z}_{{t}},\bf{x}_{{t}}{\mathscr{\sim }}{\mathscr{N}}\left(C\bf{x}_{{t}}+\bf{d},S\right)$$

Here we assume that the observation parameters *C*, **d** and *S* do not depend on *z*_*t*_.

### Sticky HDP

A key feature of depth Moseq is the use of a sticky-HDP prior for the transition matrix. In general, HDP priors allow the number of distinct states in a HMM to be inferred directly from the data. The ‘sticky’ variant of the HDP prior includes an additional hyperparameter *κ* that tunes the frequency of self-transitions in the discrete state sequence *z*_*t*_, and thus the distribution of syllable durations. As in depth MoSeq, we implement a sticky-HDP prior using the weak limit approximation^49^, as shown below:

$$\begin{array}{ll}\beta & \sim \text{Dir}\left(\gamma /N,\ldots ,\gamma /N\right)\\ {\pi }_{{i}}{\rm{| }}\beta & \sim \text{Dir}\left(\alpha {\beta }_{1},\ldots ,\alpha {\beta }_{{v}}+\kappa \ldots ,\alpha {\beta }_{{N}}\right)\end{array}$$

where *κ* is being added in the *i*th position. Here $\beta \in {\Delta }^{\rm{N}}$ is a global vector of augmented syllable transition probabilities, and the hyperparameters *γ*, *α* and *κ* control the sparsity of states, the weight of the sparsity prior and the bias toward self-transitions, respectively.

### SLDS for postural dynamics

Keypoint coordinates reflect not only the pose of an animal, but also its location and heading. To disambiguate these factors, we define a canonical, egocentric reference frame in which the postural dynamics are modeled. The canonically aligned poses are then transformed into global coordinates using explicit centroid and heading variables that are learned by the model.

Concretely, let ${Y}_{\rm{t}}\in {{\mathbb{R}}}^{\rm{K\times D}}$ represent the coordinates of *K* keypoints at time *t*, where $D\in \{2,3\}$. We define latent variables $\bf{v}_{t}\in {{\mathbb{R}}}^{D}$ and ${h}_{{t}}\in \left[0,2\pi \right]$ to represent the animal’s centroid and heading angle. We assume that each heading angle *h*_*t*_ has an independent, uniform prior and that the centroid is autocorrelated as follows:

$$\begin{array}{c}{h}_{{t}}\sim {\rm{Unif}}(0,2\pi )\\ \bf{v}_{{t}}| \bf{v}_{{t-1}}\sim {\mathscr{N}}\left(\bf{v}_{{t-1}},{\sigma }_{{\rm{loc}}}^{2}\right)\end{array}$$

At each time point *t*, the pose *Y*_*t*_ is generated via rotation and translation of a centered and oriented pose ${\widetilde{Y}}_{{t}}$ that depends on the current continuous latent state **x**_*t*_:

$${Y}_{{t}}={\tilde{Y}}_{{t}}R({h}_{{t}})+{{\bf{1}}}_{K}\bf{v}_{\rm{t}}^{\top }\,{\rm{where\; vec}}({\tilde{Y}}_{{t}})\sim {\mathscr{N}}((\varGamma \otimes {I}_{D})(C\bf{x}_{{t}}+\bf{d}),{S}_{{t}})$$

where *R*(*h*_*t*_) is a matrix that rotates by angle *h*_*t*_ in the *xy* plane, and $\varGamma \in {R}^{{K\times \left(K-1\right)}}$ is defined by the truncated singular value decomposition $\varGamma \Delta {\varGamma }^{{\rm{\top }}}={I}_{{K}}-{{\bf{1}}}_{{K\times K}}/K$. Note that *Γ* encodes a linear transformation that isometrically maps ${{\mathbb{R}}}^{{\left(K-1\right)\times D}}$ to the set of all centered keypoint arrangements in ${{\mathbb{R}}}^{{K\times D}}$, and thus ensures that ${\mathbb{E}}\left({\widetilde{Y}}_{{t}}\right)$ is always centered^50^. The parameters $C\in {{\mathbb{R}}}^{{\left(K-1\right)D\times M}}$ and $\bf{d}\in {{\mathbb{R}}}^{{\left(K-1\right)D}}$ are initialized using PCA applied to the transformed keypoint coordinates ${\varGamma }^{{T}}{\widetilde{Y}}_{{t}}$. In principle *C* and **d** can be adjusted further during model fitting, and we describe the corresponding Gibbs updates in the inference section below. In practice, however, we keep *C* and **d** fixed to their initial values when fitting keypoint-MoSeq.

### Robust observations

To account for occasional large errors during keypoint tracking, we use the heavy-tailed Student’s *t*-distribution, which corresponds to a normal distribution whose variance is itself a random variable. Here, we instantiate the random variances explicitly as a product of two parameters: a baseline variance *σ*_*k*_ for each keypoint and a time-varying scale *s*_*t,k*_. We assume:

$$\begin{array}{cc}{\sigma }_{k}^{2} & \sim {\chi }^{-2}\left({\nu }_{\sigma },{\sigma }_{0}^{2}\right)\\ {s}_{t,k}^{2} & \sim {\chi }^{-2}\left({\nu }_{s},{s}_{0,t,k}\right)\end{array}$$

where *ν*_*σ*_ > 0 and *ν*_*s*_ > 0 are degrees of freedom, ${\sigma }_{0}^{2} > 0$ is a baseline scaling parameter, and ${s}_{0,t,k} > 0$ is a local scaling parameter, which encodes a prior on the scale of error for each keypoint on each frame. Where possible, we calculated the local scaling parameters as a function of the neural network confidences for each keypoint. The function was calibrated using the empirical relationship between confidence values and error sizes. The overall noise covariance *S*_*t*_ is generated from *σ*_*k*_ and *s*_*t,k*_ as follows:

$${S}_{t}={\rm{diag}}\left({\sigma }_{1}^{2}{s}_{t,1}^{2},\ldots ,{\sigma }_{K}^{2}{s}_{t,K}^{2}\right)\otimes {I}_{D}$$

### Related work

Keypoint-MoSeq extends the model used in depth MoSeq^16^, where a low-dimensional pose trajectory *x*_*t*_ (derived from egocentrically aligned depth videos) is used to fit an AR-HMM with a transition matrix *π*, autoregressive parameters *A*_*i*_, **b**_*i*_ and *Q*_*i*_ and discrete states *z*_*t*_ like those described here. Indeed, conditional on **x**_*t*_, the models for keypoin-MoSeq and depth MoSeq are identical. The main differences are that keypoint-MoSeq treats **x**_*t*_ as a latent variable (that is, updates it during fitting), includes explicit centroid and heading variables, and uses a robust noise model.

Disambiguating poses from position and heading is a common task in unsupervised behavior algorithms, and researchers have adopted a variety of approaches. VAME^13^, for example, isolates pose by centering and aligning data ahead of time, whereas B-SOiD^12^ transforms the keypoint data into a vector of relative distances and angles. The statistical pose model GIMBAL^29^, on the other hand, introduces latent heading and centroid variables that are inferred simultaneously with the rest of the model. Keypoint-MoSeq adopts this latter approach, which can remove spurious correlations between egocentric features that can arise from errors in keypoint localization.

### Inference algorithm

Our full model contains latent variables **v**, *h*, **x**, *z* and *s* and parameters *A*, **b**, *Q*, *C*, **d**, *σ*, *β* and *π*. We fit each of these variables—except for *C* and **d**—using Gibbs sampling, in which each variable is iteratively resampled from its posterior distribution conditional on the current values of all the other variables. The posterior distributions *P*(*π*, *β*∣*z*) and *P*(*A*, *b*, *Q*∣*z*, *x*) are unchanged from the original MoSeq paper and will not be be reproduced here (see ref. ^16^, pages 42–44, and note the changes of notation *Q* → *Σ*, *z* → *x* and **x** → **y**). The Gibbs updates for variables *C*, **d**, σ, *s*, **v** and *h* are described below.

#### Resampling *P*(*C*, *d*∣*s*, *σ*, *x*, *v*, *h*, *Y*)

Let ${\tilde{\bf{x}}}_{\rm{t}}$ represent **x**_*t*_ with a 1 appended and define

$${\tilde{S}}_{t}=({\varGamma }^{\top }{\rm{diag}}(\mathop{\sigma}\nolimits_{1}^{2}{s}_{t,1},\ldots ,\mathop{\sigma }\nolimits_{K}^{2}{s}_{t,K})\varGamma )\otimes {I}_{D}$$

The posterior update is $\left(C,{\bf{d}}\right){\sim}{\mathscr{N}}\left({\text{vec}}\left(C,{\bf{d}}\right){{|}}{\mu }_{n},{\varSigma }_{n}\right)$ where

$${\varSigma }_{n}={({\sigma }_{C}^{-2}I+{S}_{x,x})}^{-1}{\rm{and}}\,{\mu }_{n}={\varSigma }_{n}{S}_{y,x}$$

with

$$\begin{array}{r}{S}_{x,x}=\mathop{\sum }\limits_{t=1}^{T}{\tilde{\bf{x}}}_{t}{\tilde{\bf{x}}}_{t}^{\top }\otimes {\varGamma }^{\top}{\tilde{S}}_{t}^{-1}\varGamma \otimes {I}_{D}\,{\rm{and}}\,{S}_{y,x}=\mathop{\sum }\limits_{t=1}^{T}\left({\tilde{\bf{x}}}_{t}^{\top }\otimes {\tilde{S}}^{-1}\varGamma \otimes {I}_{D}\right){\rm{vec}}{({\tilde{Y}}_{t})}^{\top}\end{array}$$

#### Resampling *P*(*s*∣*C*, *d*, *σ*, *x*, *v*, *h*, *Y*)

Each *s*_*t,k*_ is conditionally independent with posterior

$$\begin{array}{c}{s}_{t,k}{\rm{| }}C,\bf{d},{\sigma }_{k},\bf{x},Y\sim {\chi }^{\,-2}\left({\nu }_{s}+D,\left({\nu }_{s}{s}_{0}+{\sigma }_{k}^{-2}\parallel\Big(\varGamma {\left(C\bf{x}_{t}+\bf{d}\right)\Big)}_{k}-{\widetilde{Y}}_{t,k}{\parallel }^{2}\right)/\left({\nu }_{s}+D\right)\right)\end{array}$$

#### Resampling *P*(*σ*∣*C*, *d*, *s*, *x*, *v*, *h*, *Y*)

Each *σ*_*k*_ is conditionally independent with posterior

$$\begin{array}{c}{\sigma }_{k}^{2}\sim {\chi }^{-2}\left({\nu }_{\sigma }+{DT},\left({\nu }_{\sigma }{\sigma }_{0}^{2}+{S}_{y}\right){\left({\nu }_{\sigma }+{DT}\right)}^{-1}\right)\end{array}$$

where $${S}_{y}={\sum }_{t=1}^{N}{\Vert \varGamma {(C\bf{x}_{t}+\bf{d})}_{k}-{\tilde{Y}}_{t,k}\Vert }^{2}/{s}_{t,k}$$

#### Resampling *P*(*v*∣*C*, *d*, *σ*, *s*, *x*, *h*, *Y*)

Because the translations **v**_1_, …, **v**_T_ form an LDS, they can be updated by Kalman sampling. The observation potentials have the form ${\mathscr{N}}\left(\bf{v}_{t}{\rm{| }}\mu ,{\gamma }^{2}{I}_{D}\right)$ where

$$\mu =\sum _{k}\frac{{\gamma }_{t}^{2}}{{\sigma }_{k}^{2}{s}_{t,k}}[{Y}_{t,k}-R{\left({h}_{t}\right)}^{{\rm{\top }}}\varGamma {\left(C\bf{x}_{t}+\bf{d}\right)}_{k}],\frac{1}{{\gamma }_{t}^{2}}=\sum _{k}\frac{1}{{\sigma }_{k}^{2}{s}_{t,k}}$$

#### Resampling *P*(*h*∣*C*, *d*, *σ*, *s*, *x*, *v*, *Y*)

The posterior of *h*_*t*_ is the von-Mises distribution $\text{vM}$(*θ*, *κ*) where *κ* and $\theta \in \left[0,2\pi \right]$ are the unique parameters satisfying $\left[\kappa \cos \left(\theta \right),\kappa \sin \left(\theta \right)\right]=\left[{S}_{1,1}+{S}_{2,2},{S}_{1,2}-{S}_{2,1}\right]$ for

$$\begin{array}{c}S=\mathop{\sum }\limits_{k}\frac{1}{{s}_{t,k}{\sigma }_{k}^{2}}\varGamma {\left(C\bf{x}_{t}+\bf{d}\right)}_{k}{\left({Y}_{t,k}-\bf{v}_{t}\right)}^{{\rm{\top }}}\end{array}$$

#### Resampling *P*(*x*∣*C*, *d*, *σ*, *s*, *v*, *h*, *Y*)

To resample **x**, we first express its temporal dependencies as a first-order autoregressive process, and then apply Kalman sampling. The change of variables is

$$\begin{array}{r}A^{\prime} =\left[\begin{array}{ccccc} & I & & & \\ & & I & & \\ & & & I & \\ {A}_{1} & {A}_{2} & \ldots & {A}_{L} & \bf{b}\end{array}\right] \; Q^{\prime} =\left[\begin{array}{cccc}0 & & & \\ & 0 & & \\ & & 0 & \\ & & & Q\end{array}\right] \; C^{\prime} =\left[\begin{array}{cc}0 & 0\\ \vdots & \vdots \\ 0 & 0\\ C & \bf{d}\end{array}\right]\; \bf{x}_{t}^{\prime} =\left[\begin{array}{c}\bf{x}_{t-L+1}\\ \vdots \\ \bf{x}_{t}\\ 1\end{array}\right]\end{array}$$

Kalman sampling can then be applied to the sample the conditional distribution

$$\begin{array}{r}P({\bf{x}^{\prime}}_{1:T}| {\tilde{Y}}_{1:T})\propto \mathop{\prod }\limits_{t=1}^{T}{\mathscr{N}}({\bf{x}^{\prime} }_{t}| {A^{\prime} }^{({z}_{t})}{\bf{x}^{\prime} }_{t-1},{Q^{\prime} }^{({z}_{t})}){\mathscr{N}}({\rm{vec}}({\tilde{Y}}_{t})| {C^\prime} {\bf{x}^{\prime} }_{t},{S}_{t}).\end{array}$$

(Assume **x**′ is left-padded with zeros for negative time indices.)

### Hyperparameters

We used the following hyperparameter values throughout the paper.

#### Transition matrix

$$\begin{array}{l}N=100\\ \gamma =1,000\\ \alpha =100\\ \kappa \ \ {\rm{fit \; to \; each \; dataset}}\end{array}$$

#### Autoregressive process

$$\begin{array}{l}M \ \ {\rm{set \; using \; PCA \; explained \; variance \; curve}}\\ L=3\\ {\nu }_{0}=M+2\\ {S}_{0}=0.01{I}_{M}\\ {M}_{0}=[{0}_{M\times (L-1)}\,{I}_{M}\,{1}_{M\times 1}]\\ {K}_{0}=10{I}_{M(L+1)}\end{array}$$

#### Observation process

$$\begin{array}{l}{\sigma }_{0}^{2}=1\\ {\nu }_{\sigma }={10}^{5}\\ {\nu }_{s}=5\\ {s}_{0,t,k}\,{\rm{set}}\;{\rm{based}}\;{\rm{on}}\;{\rm{neural}}\;{\rm{network}}\;{\rm{confidence}}\end{array}$$

#### Centroid autocorrelation

$$\begin{array}{c}{\sigma }_{\text{loc}}^{2}=0.4\end{array}$$

### Derivation of Gibbs updates

#### Derivation of *C*, *d* updates

To simply notation, define

$$\begin{array}{r}{\tilde{S}}_{t}={\rm{diag}}({\sigma }_{1}^{2}{s}_{t,1},\ldots ,{\sigma }_{K}^{2}{s}_{t,K}),\,{\tilde{\bf{x}}}_{t}=(\bf{x}_{t},1),\,\tilde{C}=(C,\bf{d})\end{array}$$

The likelihood of the centered and aligned keypoint locations $\tilde{Y}$ can be expanded as follows

$$\begin{array}{ll} & P\left(\tilde{Y}{\rm{| }}\tilde{C},\tilde{\bf{x}},\tilde{S}\right)=\mathop{\prod }\limits_{t=1}^{T}{\mathscr{N}}\left(\text{vec}\left({\tilde{Y}}_{t}\right){\rm{| }}\left(\varGamma \otimes {I}_{D}\right)\tilde{C}{\tilde{\bf{x}}}_{t},{\tilde{S}}_{t}\otimes {I}_{D}\right)\\ \propto & \exp \left[-\frac{1}{2}\mathop{\sum }\limits_{t=1}^{T}\left({\tilde{\bf{x}}}_{t}^{{\rm{\top }}}{\tilde{C}}^{{\rm{\top }}}\left({\varGamma }^{{\rm{\top }}}{\tilde{S}}_{t}^{-1}\varGamma \otimes {I}_{D}\right)\tilde{C}{\tilde{\bf{x}}}_{t}-2\text{vec}{\left({\tilde{Y}}_{t}\right)}^{{\rm{\top }}}\left({\tilde{S}}_{t}^{-1}\varGamma \otimes {I}_{D}\right)\tilde{C}{\tilde{\bf{x}}}_{t}\right)\right]\\ \propto & \exp \left[-\frac{1}{2}\mathop{\sum }\limits_{t=1}^{T}\left(\text{vec}{\left(\tilde{C}\right)}^{\top }\left({\tilde{\bf{x}}}_{t}{\tilde{\bf{x}}}_{t}^{\top }\otimes {\varGamma }^{\top }{\tilde{S}}_{t}^{-1}\varGamma \otimes {I}_{D}\right)\text{vec}\left(\tilde{C}\right)\right)\right.\\ &\quad\,\, \left.\left(-2\text{vec}{\left(\tilde{C}\right)}^{{\rm{\top }}}\left({\tilde{\bf{x}}}_{t}^{{\rm{\top }}}\otimes {\tilde{S}}_{t}^{-1}\varGamma \otimes {I}_{D}\right)\text{vec}\left({\tilde{Y}}_{t}\right)\right)\,\right]\\ \propto & \exp \left[-\frac{1}{2}\left(\text{vec}{\left(\tilde{C}\right)}^{\top }{S}_{x,x}\text{vec}\left(\tilde{C}\right)-2\text{vec}{\left(\tilde{C}\right)}^{\top }{S}_{x,y}\right)\right]\end{array}$$

where

$$\begin{array}{c}{S}_{x,x}=\mathop{\sum }\limits_{t=1}^{T}{\tilde{\bf{x}}}_{t}{\tilde{\bf{x}}}_{t}^{{\rm{\top }}}\otimes {\varGamma }^{{\rm{\top }}}{\tilde{S}}_{t}^{-1}\varGamma \otimes {I}_{D}\;\text{and}\;{S}_{x,y}=\mathop{\sum }\limits_{t=1}^{T}\left({\tilde{\bf{x}}}_{t}^{{\rm{\top }}}\otimes {\tilde{S}}^{-1}\varGamma \otimes {I}_{D}\right)\text{vec}\left({\tilde{Y}}_{t}\right)\end{array}$$

Multiplying by the prior $\text{vec}\left(\tilde{C}\right){\mathscr{\sim }}{\mathscr{N}}\left(0,{\sigma }_{C}^{2}I\right)$ yields

$$\begin{array}{r}P(\tilde{C}| \tilde{Y},\tilde{\bf{x}},\tilde{S})\propto {\mathscr{N}}({\rm{vec}}(\tilde{C})| {\mu }_{n},{\varSigma }_{n})\end{array}$$

where

$$\begin{array}{cc}{\varSigma }_{n}={\left({\sigma }_{C}^{-2}I+{S}_{x,x}\right)}^{-1} \; \text{and} \; {\mu }_{n}={\varSigma }_{n}{S}_{y,x}\end{array}$$

#### Derivation of *σ*_*k*_, *s*_*t*,*k*_ updates

For each time *t* and keypoint *k*, let ${\bar{Y}}_{t,k}=\varGamma \left(C\bf{x}_{t}+\bf{d}\right)$. The likelihood of the centered and aligned keypoint location ${\tilde{Y}}_{t,k}$ is

$$P({\tilde{Y}}_{t,k}| {\bar{Y}}_{t,k},{s}_{t,k},{\sigma }_{k})={\mathscr{N}}({\tilde{Y}}_{t,k}| {\bar{Y}}_{t,k},\,{\sigma }_{k}^{2}{s}_{t,k}{I}_{D})\propto {({\sigma }_{k}^{2}{s}_{t,k})}^{-D/2}\exp \left[-\frac{{\Vert {\tilde{Y}}_{t,k}-{\bar{Y}}_{t,k}\Vert }^{2}}{2{\sigma }_{k}^{2}{s}_{t,k}}\right]$$

We can then calculate posteriors $P\left({s}_{t,k}{\rm{| }}{\sigma }_{k}\right)$ and $P\left({\sigma }_{k}{\rm{| }}{s}_{t,k}\right)$ as follows

$$\begin{array}{ll}P\left({s}_{t,k}{\rm{| }}{\sigma }_{k},{\tilde{Y}}_{t,k},{\bar{Y}}_{t,k}\right) & \propto {\chi }^{\,-1}\left({s}_{t,k}{\rm{| }}{\nu }_{s},{s}_{0}\right){\mathscr{N}}\left({\tilde{Y}}_{t,k}{\rm{| }}{\bar{Y}}_{t,k},\,{\sigma }_{k}^{2}{s}_{t,k}{I}_{D}\right)\\ & \propto {s}_{t,k}^{-1-\left({\nu }_{s}+D\right)/2}\exp \left[\frac{-{\nu }_{s}{s}_{0}}{2{s}_{t,k}}-\frac{\parallel {\tilde{Y}}_{t,k}-{\bar{Y}}_{t,k}{\parallel }^{2}}{2{\sigma }_{k}^{2}{s}_{t,k}}\right]\\ & \propto {\chi }^{\,-2}\left({s}_{t,k}{\rm{| }}{\nu }_{s}+D,\left({\nu }_{s}{s}_{0}+{\sigma }_{k}^{-2}\parallel {\tilde{Y}}_{t,k}-{\bar{Y}}_{t,k}{\parallel }^{2}\right){\left({\nu }_{s}+D\right)}^{-1}\right)\end{array}$$

$$\begin{array}{ll}P\left({\sigma }_{k}{\rm{| }}{\{{s}_{t,k},{\tilde{Y}}_{t,k},{\bar{Y}}_{t,k}\}}_{t=1}^{T}\right) & \propto {\chi }^{\,-1}\left({\sigma }_{k}^{2}{\rm{| }}{\nu }_{\sigma },{\sigma }_{0}^{2}\right)\mathop{\prod }\limits_{t=1}^{T}{\mathscr{N}}\left({\tilde{Y}}_{t,k}{\rm{| }}{\bar{Y}}_{t,k},{\sigma }_{k}^{2}{s}_{t,k}{I}_{D}\right)\\ & \propto {\sigma }_{k}^{-2-{\nu }_{\sigma }-{DT}}\exp \left[\frac{-{\nu }_{\sigma }{\sigma }_{0}^{2}}{2{\sigma }_{k}^{2}}-\frac{1}{2{\sigma }_{k}^{2}}\mathop{\sum }\limits_{t=1}^{T}\frac{\parallel {\tilde{Y}}_{t,k}-{\bar{Y}}_{t,k}{\parallel }^{2}}{{s}_{t,k}}\right]\\ & \propto {\chi }^{\,-2}\left({\sigma }_{k}^{2}{\rm{| }}{\nu }_{\sigma }+{DT},\left({\nu }_{\sigma }{\sigma }_{0}^{2}+{S}_{y}\right){\left({\nu }_{\sigma }+{DT}\,\right)}^{-1}\right)\end{array}$$

where $${S}_{y}={\sum }_{t}\parallel {\tilde{Y}}_{t,k}-{\bar{Y}}_{t,k}{\parallel }^{2}/{s}_{t,k}$$

#### Derivation of *v*_*t*_ update

We assume an improper uniform prior on *v*_*t*_, hence

$$\begin{array}{ll}P\left(\bf{v}_{t}{\rm{| }}{Y}_{t}\right) & \propto P\left({Y}_{t}{\rm{| }}\bf{v}_{t}\right)P\left(\bf{v}_{t}\right)\propto P\left({Y}_{t}{\rm{| }}\bf{v}_{t}\right)\\ & {\mathscr{\propto }}{\mathscr{N}}\left(\text{vec}\left(\left({Y}_{t}-{{\bf{1}}}_{K}\bf{v}_{t}^{{\rm{\top }}}\right)R{\left({h}_{t}\right)}^{{\rm{\top }}}\right){\rm{| }}\varGamma \left(C\bf{x}_{t}+\bf{d}\right),{S}_{t}\right)\\ & =\mathop{\prod}\limits _{k}{\mathscr{N}}\left(R\left({h}_{t}\right)\left({Y}_{t,k}-\bf{v}_{t}\right){\rm{| }}\varGamma {\left(C\bf{x}_{t}+\bf{d}\right)}_{k},{s}_{t,k}{\sigma }_{k}^{2}{I}_{D}\right)\\ & =\mathop{\prod}\limits _{k}{\mathscr{N}}\left({v}_{t}{\rm{| }}{Y}_{t,k}-R{\left({h}_{t}\right)}^{{\rm{\top }}}\varGamma {\left(C\bf{x}_{t}+\bf{d}\right)}_{k},{s}_{t,k}{\sigma }_{k}^{2}{I}_{D}\right)\\ & {\mathscr{=}}{\mathscr{N}}\left(\bf{v}_{t}{\rm{| }}{\mu }_{t},{\gamma }_{t}^{2}{I}_{D}\right)\end{array}$$

where

$$\begin{array}{c}\mu =\mathop{\sum }\limits_{k}\frac{{\gamma }_{t}^{2}}{{\sigma }_{k}^{2}{s}_{t,k}}\left({Y}_{t,k}-R{\left({h}_{t}\right)}^{{\rm{\top }}}\varGamma {\left(C\bf{x}_{t}+\bf{d}\right)}_{k}\right),\frac{1}{{\gamma }_{t}^{2}}=\mathop{\sum }\limits_{k}\frac{1}{{\sigma }_{k}^{2}{s}_{t,k}}\end{array}$$

#### Derivation of *h*_*t*_ update

We assume a proper uniform prior on *h*_*t*_, hence

$$\begin{array}{ll}P\left({h}_{t}{\rm{| }}{Y}_{t}\right) & \propto P\left({Y}_{t}{\rm{| }}{h}_{t}\right)P\left({h}_{t}\right)\propto P\left({Y}_{t}{\rm{| }}{h}_{t}\right)\\ & \propto \exp \left[\sum _{k}\frac{{\left({Y}_{t,k}-\bf{v}_{t}\right)}^{{\rm{\top }}}R\left({h}_{t}\right)\varGamma {\left(C\bf{x}_{t}+\bf{d}\right)}_{k}}{{s}_{t,k}{\sigma }_{k}^{2}}\right]\\ & =\exp \left[\frac{{\rm{tr}}\left[R\left({h}_{t}\right)\varGamma {\left(C\bf{x}_{t}+\bf{d}\right)}_{k}{\left({Y}_{t,k}-\bf{v}_{t}\right)}^{{\rm{\top }}}\right]}{{s}_{t,k}{\sigma }_{k}^{2}}\right]\\ & \propto {\rm{exptr}}\left[R\left({h}_{t}\right)S\right] \; \text{where} \; S=\mathop{\sum }\limits_{k}\varGamma {\left(C\bf{x}_{t}+\bf{d}\right)}_{k}{\left({Y}_{t,k}-\bf{v}_{t}\right)}^{{\rm{\top }}}/\left({s}_{t,k}{\sigma }_{k}^{2}\right)\\ & \propto \exp \left[\cos \left({h}_{t}\right)\left({S}_{1,1}+{S}_{2,2}\right)+\sin \left({h}_{t}\right)\left({S}_{1,2}-{S}_{2,1}\right)\right]\end{array}$$

Let $\left[\kappa \cos \left(\theta \right),\kappa \sin \left(\theta \right)\right]$ represent $\left[{S}_{1,1}+{S}_{2,2},{S}_{1,2}-{S}_{2,1}\right]$ in polar coordinates. Then

$$\begin{array}{cc}P\left({Y}_{t}{\rm{| }}{h}_{t}\right) & \propto \exp \left[\kappa \cos \left({h}_{t}\right)\cos \left(\theta \right)+\sin \left({h}_{t}\right)\sin \left(\theta \right)\right]\\ & =\exp \left[\kappa \cos \left({h}_{t}-\theta \right)\right]\propto \text{vM}\left({h}_{t}{\rm{| }}\theta ,\kappa \right)\end{array}$$

### Reporting summary

Further information on research design is available in the Nature Portfolio Reporting Summary linked to this article.

## Online content

Any methods, additional references, Nature Portfolio reporting summaries, source data, extended data, supplementary information, acknowledgements, peer review information; details of author contributions and competing interests; and statements of data and code availability are available at 10.1038/s41592-024-02318-2.

### Supplementary information

Reporting Summary

Supplementary Table 1 Number of model fits for each dataset. Each row corresponds to a particular analysis (defined by a dataset and the figure(s) in which the analysis is shown) and shows the number of times each method was applied to the dataset in the context of that analysis.

Supplementary Table 2 Parameters for fitting keypoint-MoSeq. Number of PCs and values of the stickiness hyperparameter used for each dataset. The table excludes datasets and/or analyses where the stickiness was scanned over.

Supplementary Table 3 Parameter combinations tested in Extended Data Fig. 5. Each inscribed box corresponds to one parameter scan. Rows highlighted in orange represent parameters shown in Fig. 3 and used throughout the rest of the paper. Some methods have several highlighted rows because the same parameter combination arose in multiple parameter scans.

Supplementary Video 1 Example output from three different tracking methods, illustrating noise in keypoint detections.

Supplementary Video 2 Average pose trajectories for keypoint-MoSeq syllables derived from 2D open field recordings. Each trajectory includes ten evenly timed poses from 165 ms before to 500 ms after syllable onset.

Supplementary Video 3 Example syllable instances from the 2D open field dataset. For each syllable, the video shows an array of randomly selected examples. A white dot appears in each example at the moment of syllable onset and disappears when the syllable is over.

Supplementary Video 4 Average pose trajectories for keypoint-MoSeq syllables derived from 3D open field recordings, showing an *xy* projection on the left and a *xz* projection on the right. Each trajectory includes ten evenly timed poses from 165 ms before to 500 ms after syllable onset.

Supplementary Video 5 Average pose trajectories for keypoint-MoSeq syllables derived from rat motion capture data, shown in the same format as Supplementary Video 4.

Supplementary Video 6 Example fly syllables, shown in the same format as Supplementary Video 3.

## Data Availability

This study used the following publicly available datasets: CalMS21 (https://data.caltech.edu/records/s0vdx-0k302)^32^; DeepEthogram benchmark data^31^ (https://github.com/jbohnslav/deepethogram/); Rat7M (10.6084/m9.figshare.c.5295370.v3)^51^; and fly keypoint tracking (10.1038/s41592-018-0234-5). Other data raw data generated in this study have been deposited in Zenodo (10.5281/zenodo.10636983)^52^. The thermistor recordings generated for this study are not publicly available at this time as they are being used for a follow-up paper. We plan to make these data publicly accessible upon publication of the follow-up study and in the meantime will provide them upon reasonable request.

## Code availability

Software links and user support for both depth and keypoint data are available at http://www.moseq4all.org/. Data loading, project configuration and visualization are enabled through the keypoint-moseq^53^ Python library (https://github.com/dattalab/keypoint-moseq/). We also developed a stand-alone library called jax-moseq^54^ for core model inference (https://github.com/dattalab/jax-moseq/). Both libraries are freely available to the research community under an academic and non-commercial research use license. This license permits free academic and non-commercial use, explicitly prohibits redistribution and commercial use, and requires users to agree to terms including limitations on liability and indemnity. Full license details can be viewed on the respective GitHub repository pages.
