# Supplementary material for: Keypoint-MoSeq: parsing behavior by linking point tracking to pose dynamics
Source: Nat Methods. 2024 Jul 12;21(7):1329–39. doi: 10.1038/s41592-024-02318-2 (PMC11245396; doi:10.1038/s41592-024-02318-2)
Supplement: Supplementary file 1 — Reporting Summary [file 41592_2024_2318_MOESM1_ESM.pdf]

nature portfolio

Corresponding author(s): Sandeep Robert Datta
Scott W. Linderman

Last updated by author(s): Feb 14, 2024

Reporting Summary

Nature Portfolio wishes to improve the reproducibility of the work that we publish. This form provides structure for consistency and transparency in reporting. For further information on Nature Portfolio policies, see our Editorial Policies and the Editorial Policy Checklist.

Statistics

For all statistical analyses, confirm that the following items are present in the figure legend, table legend, main text, or Methods section.

n/a	Confirmed	
☐	☒	The exact sample size (*n*) for each experimental group/condition, given as a discrete number and unit of measurement
☐	☒	A statement on whether measurements were taken from distinct samples or whether the same sample was measured repeatedly
☐	☒	The statistical test(s) used AND whether they are one- or two-sided<br>*Only common tests should be described solely by name; describe more complex techniques in the Methods section.*
☒	☐	A description of all covariates tested
☐	☒	A description of any assumptions or corrections, such as tests of normality and adjustment for multiple comparisons
☐	☒	A full description of the statistical parameters including central tendency (e.g. means) or other basic estimates (e.g. regression coefficient) AND variation (e.g. standard deviation) or associated estimates of uncertainty (e.g. confidence intervals)
☐	☒	For null hypothesis testing, the test statistic (e.g. F, t, r) with confidence intervals, effect sizes, degrees of freedom and P value noted<br>*Give P values as exact values whenever suitable.*
☐	☒	For Bayesian analysis, information on the choice of priors and Markov chain Monte Carlo settings
☒	☐	For hierarchical and complex designs, identification of the appropriate level for tests and full reporting of outcomes
☐	☒	Estimates of effect sizes (e.g. Cohen's *d*, Pearson's *r*), indicating how they were calculated

Our web collection on statistics for biologists contains articles on many of the points above.

Software and code

Policy information about availability of computer code

Data collection	Custom code for data acquisition and modeling was written in Python (details in Methods) and is available on github.<br>Acquisition code: https://github.com/calebweinreb/top-bottom-moseq (unversioned), https://github.com/calebweinreb/multicamera_acquisition (version 0.1.0)<br>Modeling code: https://github.com/dattalab/jax-moseq and https://github.com/dattalab/keypoint-moseq (version 0.0.0)
Data analysis	The following custom and publicly-available software packages were used, details are included in Methods section: Python (version 3.8), NumPy (version 1.24.3), Scikit-learn (version 1.2.2), PyTorch (version 1.9), Jax (version 0.3.22), SciPy (version 1.10.1), Matplotlib (version 3.7.1), Statsmodels (version 0.13.5), Motionmapperpy (version 1.0), DeepLabCut (version 2.2.1), SLEAP (version 1.2.3), BSOID (version 1.5.1), VAME (version 1.1), GIMBAL (version 0.0.1), HRNet (unversioned), LightningPose (version 0.0.4), segmentation_models_pytorch (version 0.3.3)

For manuscripts utilizing custom algorithms or software that are central to the research but not yet described in published literature, software must be made available to editors and reviewers. We strongly encourage code deposition in a community repository (e.g. GitHub). See the Nature Portfolio guidelines for submitting code & software for further information.

Data

Policy information about availability of data

All manuscripts must include a data availability statement. This statement should provide the following information, where applicable:

- Accession codes, unique identifiers, or web links for publicly available datasets
- A description of any restrictions on data availability
- For clinical datasets or third party data, please ensure that the statement adheres to our policy

This study used the following publicly available datasets: CalMS2133 (https://data.caltech.edu/records/s0vdx-0k302); DeepEthogram benchmark data32 (https://github.com/jbohnslav/deepethogram); Rat7M (https://figshare.com/collections/Rat_7M/5295370); Fly keypoint tracking (https://doi.org:10.1038/s41592-018-0234-5). Other data raw data generated in this study have been deposited on Zenodo (https://zenodo.org/records/10636983). The thermistor recordings are available from the authors upon reasonable request.

Human research participants

Policy information about studies involving human research participants and Sex and Gender in Research.

Reporting on sex and gender	*Use the terms sex (biological attribute) and gender (shaped by social and cultural circumstances) carefully in order to avoid confusing both terms. Indicate if findings apply to only one sex or gender; describe whether sex and gender were considered in study design whether sex and/or gender was determined based on self-reporting or assigned and methods used. Provide in the source data disaggregated sex and gender data where this information has been collected, and consent has been obtained for sharing of individual-level data; provide overall numbers in this Reporting Summary. Please state if this information has not been collected. Report sex- and gender-based analyses where performed, justify reasons for lack of sex- and gender-based analysis.*
Population characteristics	*Describe the covariate-relevant population characteristics of the human research participants (e.g. age, genotypic information, past and current diagnosis and treatment categories). If you filled out the behavioural & social sciences study design questions and have nothing to add here, write "See above."*
Recruitment	*Describe how participants were recruited. Outline any potential self-selection bias or other biases that may be present and how these are likely to impact results.*
Ethics oversight	*Identify the organization(s) that approved the study protocol.*

Note that full information on the approval of the study protocol must also be provided in the manuscript.

Field-specific reporting

Please select the one below that is the best fit for your research. If you are not sure, read the appropriate sections before making your selection.

☒ Life sciences ☐ Behavioural & social sciences ☐ Ecological, evolutionary & environmental sciences

For a reference copy of the document with all sections, see nature.com/documents/nr-reporting-summary-flat.pdf

Life sciences study design

All studies must disclose on these points even when the disclosure is negative.

Sample size	Sample sizes were not pre-determined. The number of samples (usually N=20 model runs) was chosen based on computational tractability. In practice, this sample size was easily large enough to reveal statistical significance, given the effect sizes demonstrated in the study.
Data exclusions	Recording sessions were excluded when the mice did move for the majority of the recording, i.e. of they remained in the same quadrant of the arena for >80% of frames. Thermistor recordings were excluded when the thermistor signal was excessively noisy.
Replication	Every model was run multiple times with different random seeds. No model runs were excluded.
Randomization	There were no treatment groups in our study, thus randomization was not required.
Blinding	Analysis of results did not depend on human observers, thus blinding was not necessary.

Reporting for specific materials, systems and methods

We require information from authors about some types of materials, experimental systems and methods used in many studies. Here, indicate whether each material, system or method listed is relevant to your study. If you are not sure if a list item applies to your research, read the appropriate section before selecting a response.

Materials & experimental systems

n/a	Involved in the study
☒	☐ Antibodies
☒	☐ Eukaryotic cell lines
☒	☐ Palaeontology and archaeology
☐	☒ Animals and other organisms
☒	☐ Clinical data
☒	☐ Dual use research of concern

Methods

n/a	Involved in the study
☒	☐ ChIP-seq
☒	☐ Flow cytometry
☒	☐ MRI-based neuroimaging

Animals and other research organisms

Policy information about studies involving animals; ARRIVE guidelines recommended for reporting animal research, and Sex and Gender in Research

Laboratory animals	This study utilized wild-type C57 mice, both male and female, between 6-15 weeks of age, housed in reverse 12 hr dark/light cycle at approximately 50% humidity and 21 degrees C.
Wild animals	This study did not involve wild animals.
Reporting on sex	The findings do not apply to only one sex.
Field-collected samples	This study did not involve samples collected from the field.
Ethics oversight	All experimental procedures were approved by the Harvard Medical School IACUC (Protocol # 04930) and were performed in compliance with ethical regulations of Harvard University and the Guide fir Animal Care and Use of Laboratory Animals.

Note that full information on the approval of the study protocol must also be provided in the manuscript.
